# Genome-Wide Investigation and Expression Analysis of the Catalase Gene Family in Oat Plants (*Avena sativa* L.)

**DOI:** 10.3390/plants12213694

**Published:** 2023-10-26

**Authors:** Mouna Ghorbel, Ikram Zribi, Mejda Chihaoui, Ahmad Alghamidi, Khalil Mseddi, Faiçal Brini

**Affiliations:** 1 Department of Biology, College of Sciences, University of Hail, Ha’il City 81451, Saudi Arabia; a.alghamidi@uoh.edu.sa; 2 Laboratory of Biotechnology and Plant Improvement, Center of Biotechnology of Sfax, Sfax 3018, Tunisia; ikram.zribi@cbs.rnrt.tn; 3 Computer Science Departement, Applied College, University of Ha’il, Ha’il City 81451, Saudi Arabia; ma.chihaoui@uoh.edu.sa; 4 National Center for Vegetation Cover & Combating Desertification, Riyadh 13312, Saudi Arabia; 5 Department of Biology, Faculty of Science of Sfax, University of Sfax, Sfax 3000, Tunisia; khalil.mseddi@fss.u-sfax.tn

**Keywords:** antioxidant enzymes, *Avena sativa* L., bioinformatic analysis, catalase, cis-elements regulators, oxidative stress

## Abstract

Through the degradation of reactive oxygen species (ROS), different antioxidant enzymes, such as catalase (CAT), defend organisms against oxidative stress. These enzymes are crucial to numerous biological functions, like plant development and defense against several biotic and abiotic stresses. However, despite the major economic importance of *Avena sativa* around the globe, little is known about the *CAT* gene’s structure and organization in this crop. Thus, a genome-wide investigation of the *CAT* gene family in oat plants has been carried out to characterize the potential roles of those genes under different stressors. Bioinformatic approaches were used in this study to predict the *AvCAT* gene’s structure, secondary and tertiary protein structures, physicochemical properties, phylogenetic tree, and expression profiling under diverse developmental and biological conditions. A local Saudi oat variety (AlShinen) was used in this work. Here, ten *AvCAT* genes that belong to three groups (Groups I–III) were identified. All identified CATs harbor the two conserved domains (pfam00199 and pfam06628), a heme-binding domain, and a catalase activity motif. Moreover, identified AvCAT proteins were located in different compartments in the cell, such as the peroxisome, mitochondrion, and cytoplasm. By analyzing their promoters, different cis-elements were identified as being related to plant development, maturation, and response to different environmental stresses. Gene expression analysis revealed that three different *AvCAT* genes belonging to three different subgroups showed noticeable modifications in response to various stresses, such as mannitol, salt, and ABA. As far as we know, this is the first report describing the genome-wide analysis of the oat catalase gene family, and these data will help further study the roles of *catalase* genes during stress responses, leading to crop improvement.

## 1. Introduction

Environmental stresses can considerably affect crops by more than 50% and this by affecting morphological and physiological changes in plants, which can have an impact on yield in addition to genetic factors [1]. In fact, biotic and abiotic stressors have a negative impact on crop yields, biomass output, and survival [2]. In general, oxidative stress is a secondary stress caused by the generation of reactive oxygen species (ROS) that occurs during aerobic cellular processes and causes an imbalance between ROS production and removal, which increases free radicals in cells [3]. ROS are generated by different compartments in cells, specifically the peroxisomes, mitochondria, the endoplasmic reticulum, and chloroplasts. According to several studies [4,5], 1–2% of the oxygen a plant consumes creates ROS. At low concentrations, ROS fundamentally act as second messengers in intracellular signaling cascades of stress responses, whereas at high concentrations, ROS accumulation damages cells [6]. Plant antioxidant systems can regulate ROS accumulation by controlling it synthesis under normal aerobic metabolism. This redox balance is disrupted under various stress situations by high ROS levels, depletion of antioxidant defense systems, or both, causing plant cell collapse and cell death (necrosis) [7]. Biomolecules, including DNA, proteins, and lipids, can become damaged when the level of ROS surpasses the body’s ability to defend itself [4].

A significant plant adaptation method to combat oxidative stress is activating several enzymatic and non-enzymatic antioxidants [8]. In fact, a variety of enzymes are stimulated to lessen the effects of oxidative stress. These include ascorbate (APX), glutathione (GH) cycle enzymes, catalase (CAT), and superoxide dismutase (SOD). Almost all aerobic organisms have the antioxidant enzyme CAT, which has a high affinity for H_2_O_2_. In peroxisomes, hydrogen peroxide (H_2_O_2_) is converted into water and oxygen [9].

In several taxa, including bacteria and mammals, CATs have been isolated and structurally described [10]. However, a limited understanding of CAT functions in plants is available. Some data are available from different species, such as rice and *Arabidopsis* [11], bread wheat [12], durum wheat [13,14,15,16], cotton [17], sugarcane [18], cucumber [19], tobacco [20], rapeseed [21], and *Phyllanthus emblica* [22]. Plant abiotic stress tolerance may be positively correlated with CAT transcriptional activation in maize [23], *Zostera marina* [24], broccoli [25], olive [26], durum wheat [16], tobacco [20], cucumber [27], sugarcane [18,28], and banana [29].

A vital and well-established cereal crop, oats (*Avena sativa* L.) are grown essentially throughout North America and Europe [30]. Actually, oats are grown worldwide as feed because of their high protein and vital mineral content [31]. Additionally, it is a good source of dietary fiber, particularly glucan, which may benefit human health [32]. This crop is frequently grown in regions with many drawbacks, like drought or severe salt, because it is less profitable than maize, soybean, or wheat crops [33].

This species is also sensitive to light and temperature variations [34]. Because the genomes of the most significant crops, like rice [35], maize [36], and wheat [37], were sequenced, it was possible to further understand these species, but the current understanding of the stress–adaptive mechanisms in oats is still limited on a molecular level. Moreover, oats can be grown as a hay crop, cover crop, pasture plant, or green manure. It acts as a cover crop, enhancing soil health, weed control, erosion reduction, and soil organic content [38]. All of this has raised the demand for high-quality oats. Due to the high capacity of oat farming to retain salt ions in its straw biomass, it is an efficient biological method to improve salty lands [39]. 

Cultivated oat is an allohexaploid species (AACCDD, 2n  =  6x  =  42) [40] thought to have been domesticated more than 3000 years ago while growing as a weed in wheat, emmer, and barley fields in Anatolia [41]. This hexaploid species (6x = 42) adapts to several soil types and prospers best in cool, humid climates [42]. In nature, *Avena* species are found as diploids, tetraploids, and hexaploids and represent the most important genetic diversity around the Canary Islands, the Mediterranean, the Middle East, and the Himalayas [41]. Oat plants are more pH adaptable than wheat or barley, ranging from 5.5 to 7.0 and even as low as 4.5 for some ecotypes. Oats, however, need enough water for growth and grain production. Oats have long been superior to animal feed due to their high protein and important mineral content [38]. Due to disease resistance and low nutrient needs, oat plantations also have a relatively low input need for pesticides, fungicides, and fertilizers. Although oats are primarily used as animal feed now, they are one of the cereals with the greatest potential for usage in functional foods in the future.

According to Willenborg et al. [43], salinity affects seed germination, growth, water/nutrient intake, and oat plant physiological, morphological, and biochemical processes. In addition to being a significant source of dietary fiber, oats also contain a number of lipids, essential minerals, and the mixed-linkage polysaccharide-glucan. Other phytoconstituents found in oats include triterpenoids, flavonoids, saponins, flavonolignans, avenanthramides, gramine, sterols, and tocols. Oats are traditionally used for their diuretic, stimulant, anticancer, antispasmodic, and neurotonic properties. In addition, oats have a variety of pharmacological properties, including antidiabetic, anti-inflammatory, wound healing, immunomodulatory, antioxidant, and anti-cholesterolemic properties. Regarding all those benefits, oats are now grown worldwide and are a key component of many people’s diets [44].

Different cell structures, including the chloroplasts, mitochondria, and peroxisomes, are capable of producing ROS [1,2] by interfering with cellular signaling pathways, stimulating immune cells, inducing cell growth, senescence, and apoptosis, moderate quantities of H_2_O_2_ function as second messengers [3,4]. However, high concentrations of H_2_O_2_ can harm DNA, proteins, and lipids by causing oxidative stress [3,4]. Thus, ROS must be changed into less harmful forms in order to prevent such detrimental effects on cells. Plants are carried out with an effective scavenging system such as antioxidant enzymes [5]. Among those enzymes, CATs and peroxidases have a high affinity for H_2_O_2_ and can break it down into oxygen and water [6]. In peroxisomes, CATs are potent antioxidant metalloenzymes, as demonstrated by the genes identified from *Triticum durum* [7], *Triticum monococcum* [8], *Hordeum vulgare* and *Triticum turgidum* [9]. This location is under the control of a peroxisomal targeting signal type 1 (PTS1) conserved motif found at the C-terminal portion of the protein. Such domain is the most frequent peroxisome-targeting protein in plants [10]. Numerous plant species, including *Gossypium hirsutum* and *G. barbadense* [11], *Cucumis sativus* [12], rice [13], *T. aestivum* [14], *Brassica napus* [21], and *Saccharum spontaneum* [18], have had multiple *CATs* genes characterized to date. Four distinct *CAT* genes have recently been found in barley. Responses to drought stress resulted in the activation of CAT2 and CAT4 in this species [17]. Plants’ reactions to biotic and abiotic stresses are controlled by *CAT* genes [6,11,18]. Three isoforms have been found in *Arabidopsis*, and they are controlled in response to the developmental process as well as different abiotic stimuli [19,20]. In *G. hirsutum* and *G. barbadense* plants, expression analysis revealed that the *CAT* gene is involved in the plant’s response to infection with the *V. dahlia Kleb* pathogen [11], whereas in *T. turgidum* and *H. vulgare*, TtCAT, and HvCAT are induced after plant exposure to different hormones (salicylic acid (SA) and abscisic acid (ABA)) and abiotic stresses (heavy metals, heat, ionic, and osmotic stress) [9].

In monocotyledons, durum wheat catalase (TdCAT1), belonging to the subfamily I, is well studied [7,21,22]. In fact, TdCAT1 harbors different conserved domains such as the catalase domain, the peroxisome localization signal (PTS1), cations binding domains (Ca^2+^, Mn^2+^, Cu^2+^/Zn^2+^, Fe^2+,^ and Mg^2+^) located at different parts of the protein, the calmodulin-binding domain (CaMBD) [21] and an autoinhibitory domain [22] located at the C-terminal portion of the protein. In addition, it has been demonstrated that TdCAT1 confers plants’ tolerance to different abiotic stresses, such as salt, ionic, and osmotic stresses, as well as heavy metals [7,22]. To our knowledge, there is little known about the protein composition of the oat species, particularly the varieties grown in Saudi Arabia. Therefore, we conducted a comprehensive genome-wide analysis of *CAT* genes in this allohexaploid plant. In this work, ten catalase-encoding genes were identified. In fact, phylogenetic relationships, gene structures, and locations, conserved domains, in silico subcellular localization, and other analyses of *AvCAT* genes were conducted. In addition, the relative expression levels of three different *AvCAT* genes belonging to three different subgroups showed noticeable modifications in response to various stresses such as mannitol, salt, and ABA. These outcomes boost our understanding of the evolutionary history and biological function of *AvCAT* genes in oats. This study also paves the way for further analysis of the role of *CAT* gene families.

## 2. Results

### 2.1. Bioinformatic Analysis of Catalase Genes in A. sativa L.

**Figure 1:**
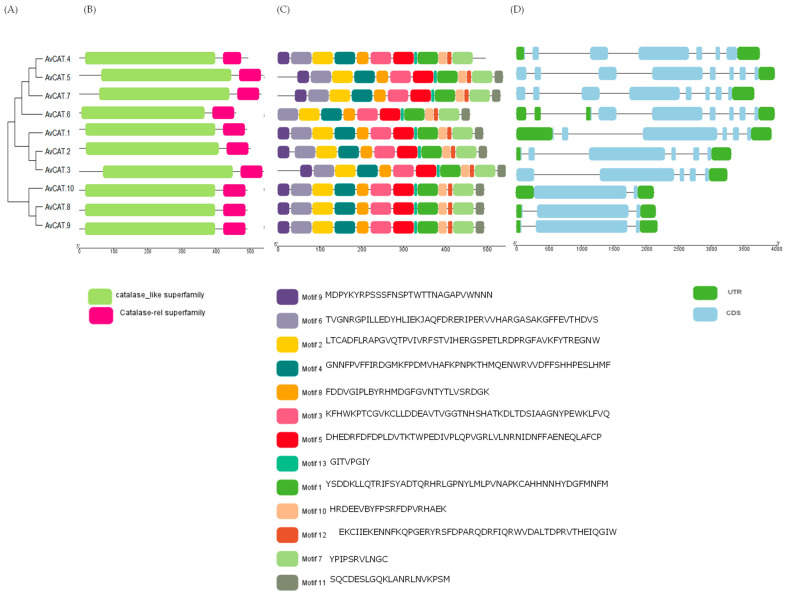
Bioinformatic analysis of AvCAT genes/proteins visualized by Tbtools. (**A**) A phylogenetic tree produced by MEGA 11 shows the phylogenetic relationship between the identified genes. (**B**) Identification of conserved catalase domains (catalase-like superfamily and catalase-related superfamily) present in AvCAT proteins as revealed by CDD online tool. (**C**) Representation of conserved motifs of AvCAT proteins as revealed by MEME server. (**D**) *AvCAT* gene’s structure. The abscissa in B, C, and D represent the length of the different proteins or genes. The blue rectangle in D represents the CDS of the genes, and the green boxes represent the UTR regions.

Firstly, we collected the gene structure information of *AvCATs* in the gene annotation file and visualized it using the web tool (Figure 1A). Ten *CAT* genes (named AvCAT1 to AvCAT10; Table 1) were identified in oats. 

**Figure 2:**
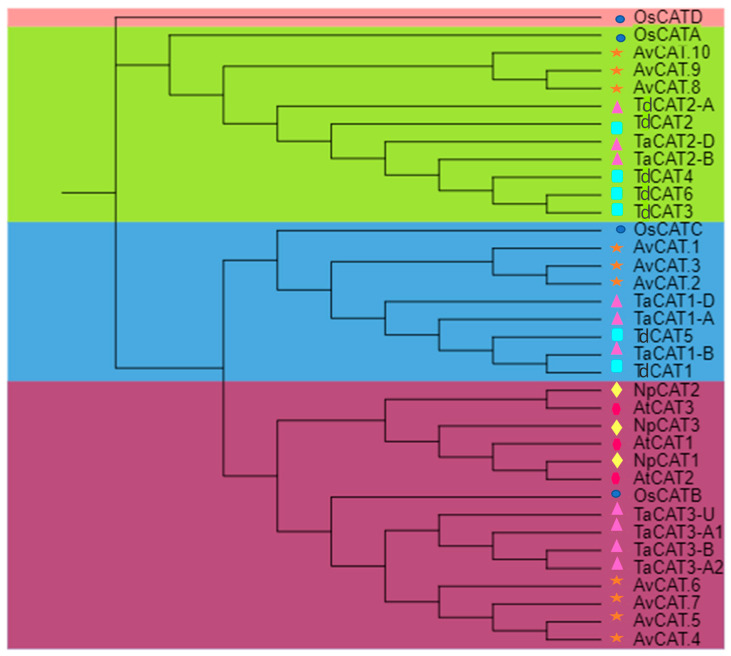
The phylogenetic tree of catalase proteins identified in different species: *A. sativa* L. (AVESA.00001b.r3.1Dg0003456.1; AVESA.00001b.r3.4Ag0002488.4; AVESA.00001b.r3.4Cg0001036.2; AVESA.00001b.r3.7Dg0000025.2; AVESA.00001b.r3.7Dg0002783.2; AVESA.00001b.r3.7Dg0002783.1; AVESA.00001b.r3.6Cg0000037.1; AVESA.00001b.r3.2Dg0000518.1; AVESA.00001b.r3.1Ag0002627.3; AVESA.00001b.r3.6Cg0001322.3); *T. turgidum* ssp durum (WDD45561.1; VAI41949.1; VAI53367.1; VAI53366.1; VAI10245.1; VAI53365.1); *O. sativa* ssp japonica (OsCATA: XP_015625395; OsCATB: XP_015643077; OsCATC: Q10S82.1; OsCATD XP_015636098.1), *A. thaliana* (AtCAT1: AAQ56816.1; AtCAT2: AAL66998.1; AtCAT3: NP_564120.1), *N. plumbaginifolia* (NpCAT1: P49315.1; NpCAT2: P49316.1; NpCAT3: P49317.1), and *T. aestivum* (TaCAT1-B: TraesCS4B02G325800; TaCAT1-D: TraesCS4D02G322700; TaCAT1-A: TraesCS5A02G498000; TaCAT2-A: TraesCS6A02G04170; TaCAT2-B: TraesCS6B02G056800; TaCAT2-D: TraesCS6D02G048300; TaCAT3-A1: TraesCS7A02G549800; TaCAT3-A2: TraesCS7A02G549900; TaCAT3-B: TraesCS7B02G473400; TaCAT3-U: TraesCSU02G105300) was constructed with test maximum likelihood with 1000 bootstraps by MEGA 11.

A phylogenetic tree was produced using the ten AvCAT proteins identified in the allohexaploid genome to investigate the evolutionary relationship between oat CAT proteins (Figure 1A and Figure 2). The phylogenetic tree showed that AvCAT proteins are subdivided into three clusters using two servers (Figure 1B; Supplemental Figure S1). AvCAT1, AvCAT2, and AvCAT3 proteins are clustered in the same group (group 1). The second group is composed of AvCAT4, AvCAT5, AvCAT6, and AvCAT7 proteins, whereas the third group is formed by AvCAT8, AvCAT9, and AvCAT10 proteins. 

Furthermore, an unrooted phylogenetic tree was constructed using MEGA 11 software with different CAT proteins from *Arabidopsis thaliana* (3 proteins), *T. aestivum* (10 proteins), *T. turgidum* (6 proteins), *Nicotiana plumbaginifolia* (3 proteins); and *Oryza sativa* ssp Japonica (4 proteins) (Figure 2). As represented by the phylogenetic tree, thirty-six *CAT* genes were divided into three different classes (class I, class II, and class III), with an extra group formed exclusively by OsCATD. The dicotyledonous CAT proteins identified in *A. thaliana* and *N. plumbaginifolia* were clustered in the same group together with oat CAT belonging to the second group (AvCAT4/5/6 and 7), OsCATB and TaCAT3 (A1/A2/B/U) and no CAT from *T. turgidum* suggesting a related relationship between those proteins. The other two groups (groups 1 and 3) are formed exclusively by monocotyledonous CATs. Group 1 is composed of Avena CAT group 3 (AvCAT8, 9 and 10), TaCAT2 (genome A/B/D), OsCATA/D, and TdCAT2/3/4 and 6, which suggests that those CAT proteins could share the same functions in plants. The last group is formed by Avena CAT class 1, TdCAT1/5, TaCAT1A/B/D, and OsCATC (Figure 2).

Subsequently, the conserved domain of candidate AvCAT protein sequences was analyzed (Figure 1B). The multiple alignments performed using the Muscle algorithm showed that AvCAT protein sequences are conserved (Supplemental Figure S2). Based on the domain analysis, all identified AvCAT proteins contained one catalase core domain (PF00199, Catalase) and one catalase immune-responsive domain (PF06628, Catalase-rel), forming the fundamental catalase domains. The catalase-rel domain was an immune-responsive amphipathic octa-peptide that was found in the C-terminal of CATs. In addition, despite their different sizes, all CAT proteins have the same conserved domains, such as pfam00199 and pfam06628 domains. Moreover, as found in typical CAT proteins, all identified AvCAT proteins harbor a conserved catalase activity motif (CAM: FARERIPERVVHARGAS) site, which also presented the conserved Histidine residue at position 65 (Supplemental Figure S2). In addition, a conserved heme-binding site (HBS: RVFAYGDTQ) with a conserved Tyrosine (Y350) is also conserved in all AvCAT proteins (Supplemental Figure S2). Finally, a PTS1-like motif (QKL/I/V) was also mapped in AvCAT1/2/3/5/6/7, whereas AvCAT4 presents the CSS motif and AvCAT8/9/10 presents the MKV motif in their sequences (Supplemental Figure S2). Moreover, the histidine residue is conserved in all identified proteins (Supplemental Figure S2). 

In the second step, we used the Multiple Em for Motif Elicitation (MEME) database (version 5.5.1) to map the putative conserved motifs in the identified AvCAT proteins. As shown in Figure 1C, thirteen motifs were identified. Interestingly, those motifs are present in all identified proteins except AvCAT9, which lacks motif 9 (presented by dark purple boxes). Moreover, to understand the evolution of *AvCAT* genes, analyses of the exon–intron organization of *AvCAT* genes was performed. The result of the *AvCAT* gene structure showed that the numbers of exons varied between two and nine, with the lowest numbers of exons in AvCAT10 and the highest number in AvCAT6 (Table 1; Figure 1D). AvCAT8 and AvCAT9, which belong to the same cluster, present three exons. AvCAT3 harbors five exons, whereas AvCAT1 and 2, belonging to the same cluster, harbor six exons. The other catalases belonging to the same group harbor seven exons in AvCAT4, eight exons in AvCAT5, and seven and nine exons in AvCAT6. The genomic features of identified *AvCAT* genes are detailed in Table 1.

### 2.2. Gene Distribution of Catalase Genes in Oat

**Figure 3:**
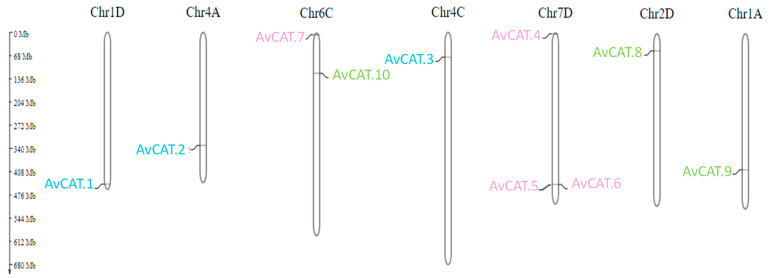
Chromosome localization of *AvCAT* genes. Prediction of AvCAT genes chromosomal localization in *A. sativa* genome using MG2C v2.0 online tool. The classification was based on their groups I, II, and III. Gene IDs are colored in blue, pink, and green, respectively.

The distribution of *CAT* genes in allohexaploid oats was observed on different chromosomes. In fact, two *CAT*-encoding genes are located in chromosome 1 (genomes A and D); one *CAT* gene is located in chromosome 2D; two *CAT*-encoding genes are located in chromosome 4 (genomes A and C); two *CATs* encoding genes are located in chromosome 6 and three *CAT* encoding genes are located in chromosome 7 (genome D) (Table 1, Figure 3).

### 2.3. AvCAT Proteins Characteristics 

All identified proteins are stable except AvCAT3 and AvCAT5. In addition, the secondary (2D) structure of AvCAT proteins was predicted using the SOPMA program. Interestingly, five proteins (AvCAT1/2/3/6 and 7) present a high aliphatic index (>70), which suggests that those proteins are thermostable over a wide temperature range. Moreover, AvCAT proteins presented small, disordered regions in their structures located at the C-terminal part of the proteins, except for AvCAT4, where the disordered region was located in the N-terminal part (Table 2). All identified proteins revealed alpha helix, beta turns, extended strand, and random coil. These structures were represented by small lines of different colors (Supplemental Figure S3; Table 2). 

**Figure 4:**
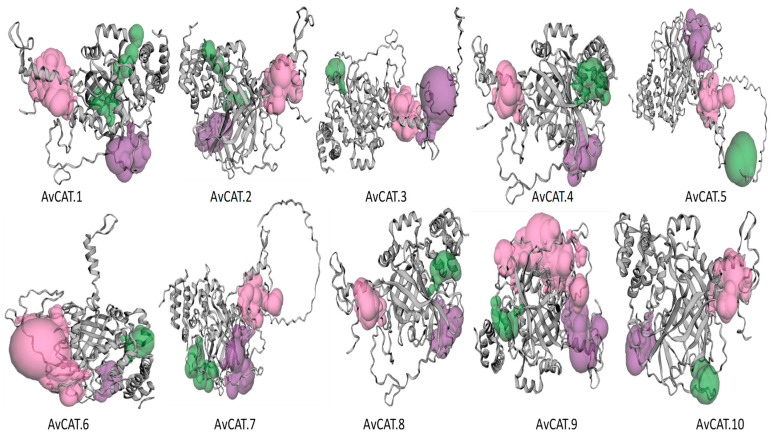
The predicted 3D structures of AvCAT were built using the SWISS-MODEL web server, and prediction of the binding pocket of catalase protein in *Avena sativa* was generated by the CASTp 3.0 online tool. Pockets were visualized from the largest to the smallest pocket with pink, purple, and green colors, respectively.

Interestingly, the organization of these secondary structures of AvCAT proteins differed. In fact, the 2D structure of AvCAT1, 2, 4, and 8 was very similar despite the fact that they belong to different sub-classes, as revealed by the phylogenetic analysis. AvCAT4 differs slightly from those proteins in the C-terminal part. Despite their similarity in the CDS organization and the motifs identified in their structures, AvCAT8 slightly differs from AvCAT9 and AvCAT10 2D structures, whereas AvCAT9 and AvCAT10 structures were more similar. The same result was obtained for AvCAT5 and AvCAT7. Finally, the AvCAT3 and AvCAT6 structures differ from each other and the other proteins (Supplemental Figure S3). Random coil accounted for a large proportion (47–54%) of all identified CAT proteins. The second was alpha-helix (26–30%), and those components were concentrated in the N-terminal region of the proteins. Beta turns formed the smallest proportion (4–6%) of secondary structures, whereas the extended strands counted between 13 and 16% (Table 2). In addition, the Alphafold server was used to predict the 3D structures of the proteins (Figure 4). The AvCAT tridimensional protein structures have some differences from each other, confirming the results already described by the 2D structure. In fact, AvCAT1, 2, 4, and 8 was very similar. AvCAT9 and AvCAT10 structures were also very similar, as revealed by the 2D structure—the same for AvCAT5 and AvCAT7. Finally, the AvCAT3 and AvCAT6 structures differ from each other and the other proteins (Figure 4).

Interestingly, all proteins’ GRAVY index (grand average of hydropathy) is negative, suggesting that those proteins are hydrophobic. Finally, all identified proteins have a high percentage of random coils (Table 3). CAT proteins do not have a signal peptide site in their structures, except for AvCAT5 (Table 3), as revealed by the Protter server. Finally, a glycosylation site was identified for all oat CAT proteins except for AvCAT5, which presented two glycosylation sites, and AvCAT6, which does not have a glycosylation site. These sites are located at different parts of the proteins (Table 3). Additionally, using NetPhos-3.1: https://services.healthtech.dtu.dk/services/NetPhos-3.1/, AvCAT proteins were analyzed to count the number of phosphorylated sites in the proteins. As expected, all AvCAT proteins are phosphorylable as the number of identified phosphorylation sites varies from 29 in AvCAT6 to 50 in AvCAT5, which suggests that protein phosphorylation is important for AvCAT protein activities (Table 3). In addition, no transmembrane region was found in all identified AvCAT proteins except for AvCAT5. 

### 2.4. In Silico Analysis of AvCAT Proteins

**Figure 5:**
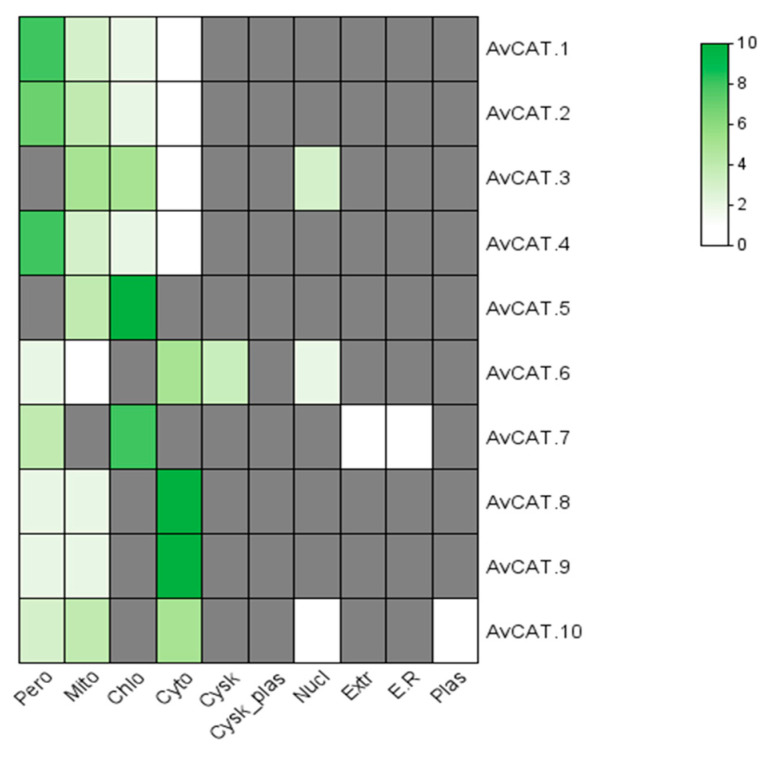
Prediction of subcellular localization of AvCAT proteins using Wolf PSORT online server and visualization via Tbtools software v1.123.

On the other hand, the subcellular localization of AvCAT proteins was performed using the Wolf PSORT online server. As shown in Figure 5, AvCAT proteins presented different subcellular localizations. In fact, no catalase protein was identified in the extracellular compartment, endoplasmic reticulum, and plasmatic membrane. AvCAT1, AvCAT2, and AvCAT4 are essentially peroxisomal proteins. AvCAT3 could be located in the mitochondria and chloroplasts. AvCAT5 and AvCAT7 are chloroplastic. AvCAT6 could be found in the peroxisome, mitochondria, and the cytoplasm. AvCAT8 and AvCAT9 are predominantly located in the cytoplasm but could also be mitochondrial and peroxisomal, whereas AvCAT10 is predicted to be a cytoplasmic, peroxisomal, and mitochondrial protein with a small probability of a nucleic localization (Figure 5).

### 2.5. Identification of CaM Binding Domains

To search whether identified oat CAT proteins harbor a calmodulin-binding domain, we analyzed the structure of the identified proteins using the calmodulin target database. As revealed in Table 4, all identified AvCAT proteins harbor at least three putative CaMBDs located at different parts of the proteins. All identified CAT proteins harbor an IQ motif (Table 4). The biological significance of such domains remains unclear.

### 2.6. Gene Ontology (GO) Term Distribution of A. sativa Catalase 

**Figure 6:**
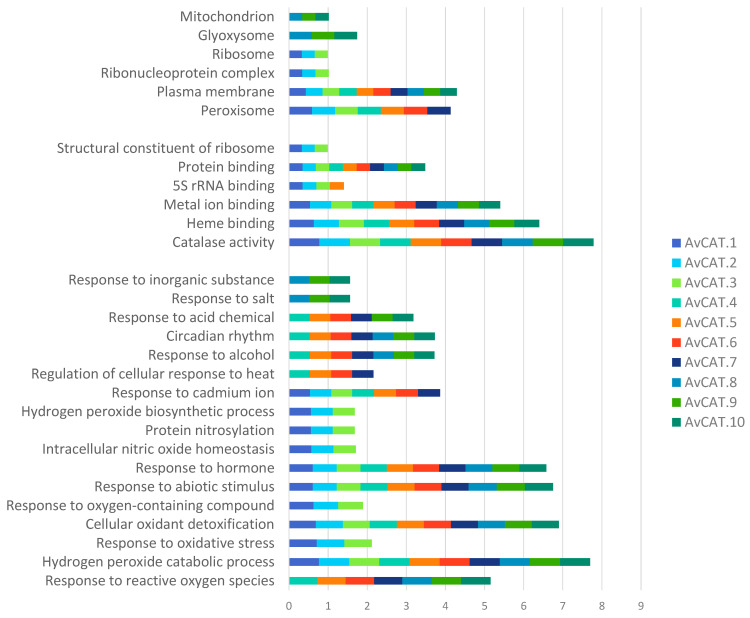
Go ontology prediction of catalase proteins of *A. sativa* plant realized by PANNZER2 webtool.

To identify the biological process and the molecular functions of the different isolated proteins, the Pannzer2 tool was used. The results, represented in Figure 6, showed that all identified proteins have a catalase activity and present heme/metal binding motifs. Additionally, AvCAT proteins present a protein-binding function. In addition, AvCAT1, 2, and 3 are structural constituents of ribosomes, whereas AvCAT1, 2, 3, and 5 have 5S rRNA binding functions (Figure 6). Interestingly, oat CATs have different functions. In fact, all identified AvCAT control the cellular oxidant detoxification and hydrogen peroxide catabolic process. Moreover, those genes are implicated in response to hormones and different abiotic stresses. AvCAT4, 5, 6, 7, 8, 9, and 10 are involved in cell response to ROS whereas, AvCAT1, 2, and 3 are involved in response to oxygen-containing substances, oxidative stress, intracellular nitric oxide homeostasis, Hydrogen peroxide biosynthesis processes and implicated in protein nitrosylation. In addition, AvCAT8, 9, and 10 control plant response to inorganic substances and salt stress. AvCAT4/5/6 and 7 control plant response to heat and, together with AvCAT4, 5, and 6, modulate plant response to cadmium. AvCAT4, 5, 6, 7, 8, 9, and 10 control the circadian rhythm of the plants and their response to alcohol stress. Finally, AvCAT4, 5, 6, 7, 9, and 10 ensure plant response to acid chemicals (Figure 6).

### 2.7. In Silico Analysis of Cis-Elements

**Figure 7:**
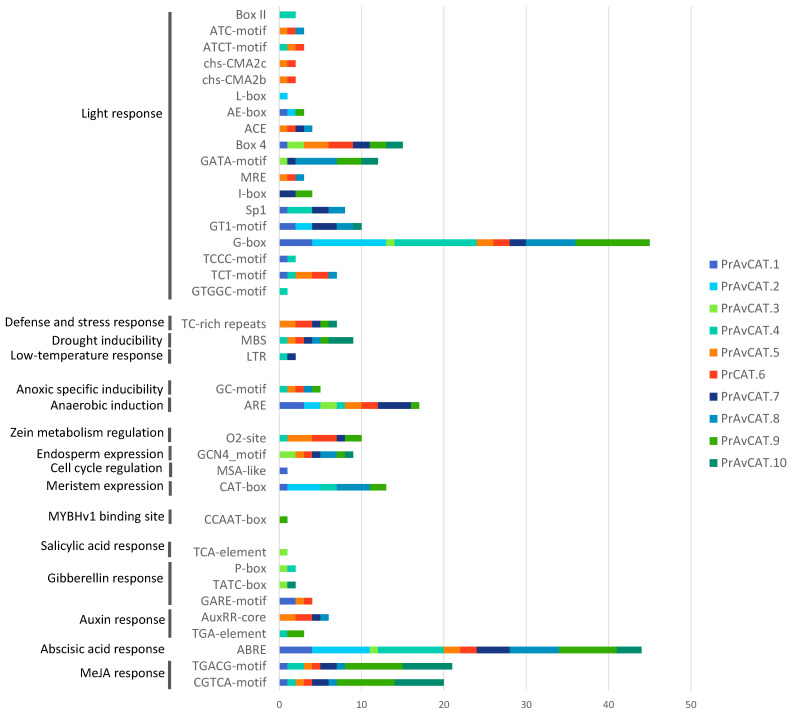
Frequency of the cis-elements in AvCAT promoters as revealed by Plantcare.

To further investigate the cis-elements of different CAT genes, the 2 kb 5′ upstream region of the 10 *AvCAT* genes was studied using the PlantCARE database. Our results showed the presence of some basic core components as well as the different cis-acting elements that could be divided into three categories: environmental stress-related elements (like drought inducibility, light-responsive and low temperature-responsive), anoxia or anaerobic induction elements, and hormone-responsive elements (such as Me-Jasmonic acid (MeJA), salicylic acid (SA), auxin, Gibberelline, and abscisic acid-responsive (ABRE)) and development-related elements (such as cell cycle regulation and meristem expression) (Figure 7). Different identified elements, such as ABRE and MeJA-response elements, are crucial for abiotic stress response. G-box and ABRE elements are common for all identified *AvCAT* genes (Figure 7). Moreover, all *AvCAT* genes are implicated in plant response to light, but only AvCAT4 and AvCAT7 are implicated in plant response to low temperatures, whereas AvCAT3 is the only *CAT* gene that presents responsive elements to SA in its promoter. Thus, *CAT* genes from oats belonging to the same sub-group could have various modes of action, and genes of different classes may work together.

### 2.8. Expression Analysis of AvCATs in Different Tissues/Organs and under Different Stress Conditions

**Figure 8:**
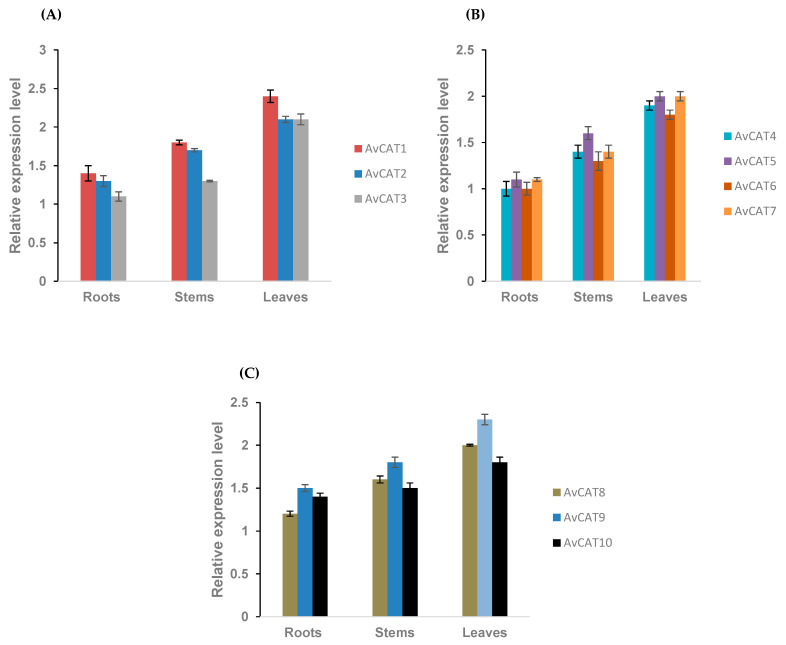
RT-qPCR expression analysis of *AvCAT* genes under normal conditions. *AvCAT* gene expression of groups 1 (**A**), 2 (**B**), and 3 (**C**) was analyzed under normal conditions using tissues from roots, leaves, and stems.

To investigate the biological functions of the CAT gene family in the allohexaploid oat plant further, we investigated the transcriptome data of different tissues/organs. In fact, in our analysis, we examined the expression pattern of *AvCAT* genes in three organs of oats (stems, roots, and shoots) at 10 days-old stage at normal conditions. As seen in Figure 8, all AvCATs have a constitutive expression in all organs. Remarkably, the *AvCAT3* gene, which belongs to group I, showed a lower expression than other genes of group I, especially in roots and leaves, whereas AvCAT1 presented a higher expression level (Figure 8).

**Figure 9:**
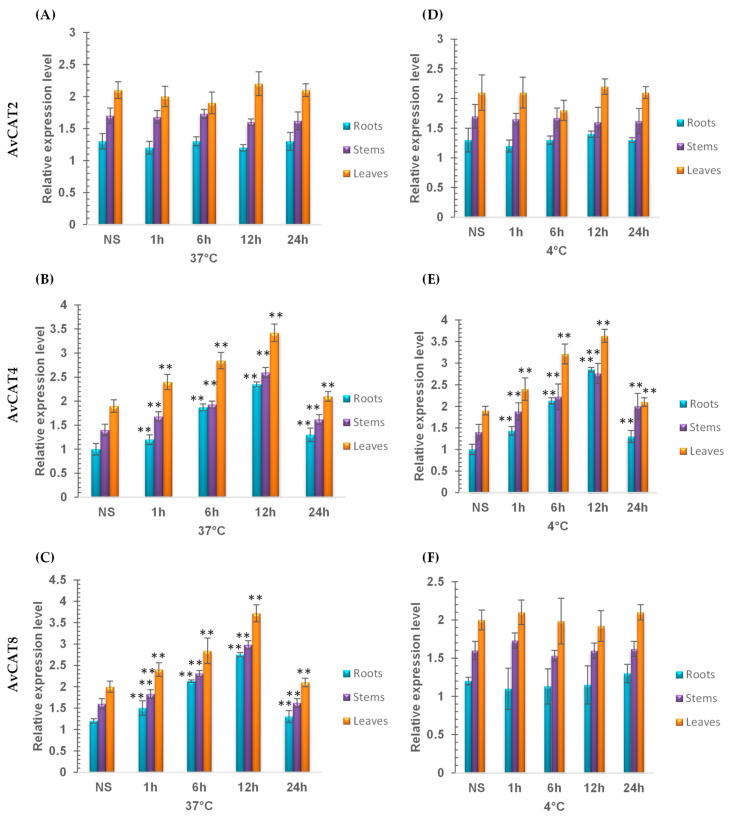
RT-qPCR expression analysis of *AvCAT2*, *AvCAT4,* and *AvCAT8* genes under heat (**A**–**C**) and cold (**D**–**F**) stress conditions and using tissues from roots, leaves, and stems. ** Indicates values significantly different from the control. Statistical significance was assessed by applying the Student’s *t*-test at *p* < 0.05.

In the second step, we chose 3 *AvCAT* genes representing three different *AvCAT* gene classes, AvCAT2, AvCAT4, and AvCAT8, to study their expression under different stress conditions. Under heat stress conditions (37 °C), AvCAT2 had no variation in gene expression, and the transcription level remained unchanged (Figure 9A). Interestingly, *AvCAT4* and *AvCAT8* gene expression started to increase after 1 h of stress application to reach their maximum after 12 h before declining after 24 h of stress (Figure 9B,C).

Under cold stress conditions, AvCAT2 and AvCAT8 did not present any modification of gene expression levels in contrast to AvCAT4, which showed an increase in its transcript level (Figure 9D–F). Such results showed the importance of AvCAT4 in plant response to heat and cold stress and the role of AvCAT8 in heat stress. Moreover, AvCAT2, a group I catalase, is not implicated in plant response to extreme temperature stress.

Our results suggest that AvCAT2 is not involved in plant defense against heat and cold stresses, whereas AvCAT4 is crucial in plant response to both stresses. Moreover, AvCAT8 is involved in heat stress response (Figure 9).

**Figure 10:**
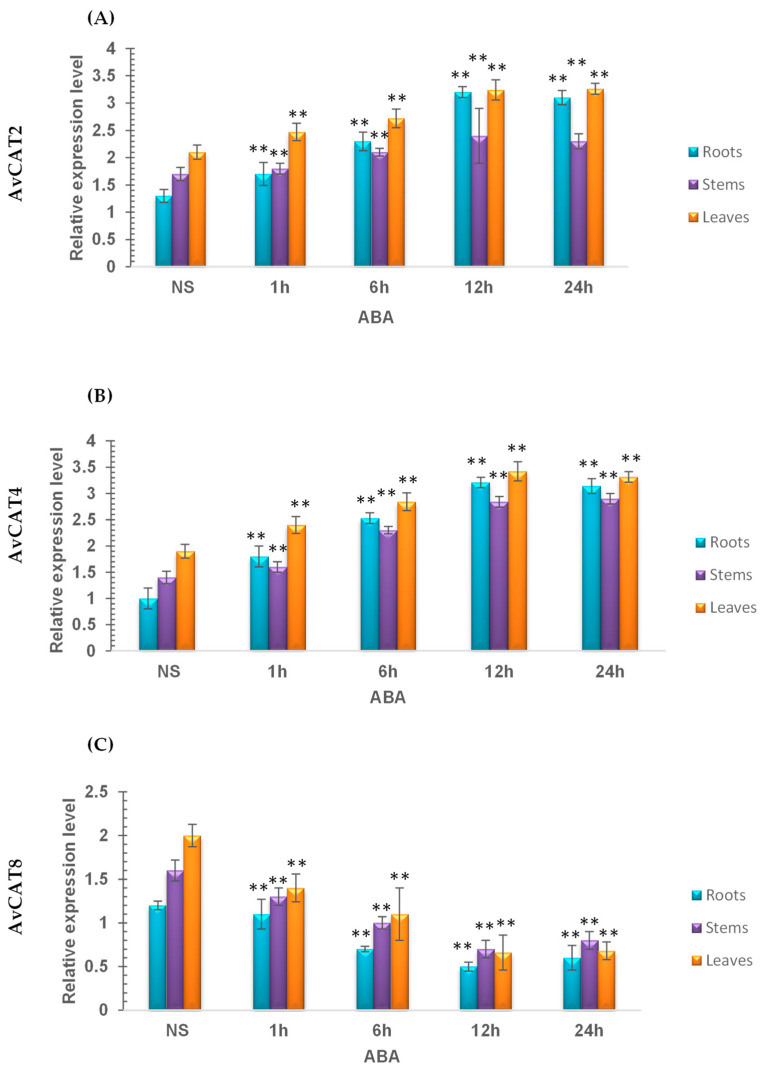
RT-qPCR expression analysis of *AvCAT2* (**A**), *AvCAT4* (**B**), and *AvCAT8* (**C**) genes under ABA stress treatment in different tissues isolated from roots, leaves, and stems. ** Indicates values significantly different from the control. Statistical significance was assessed by applying the Student’s *t*-test at *p* < 0.05.

Under ABA stress conditions, AvCAT2 was rapidly activated in all investigated tissues with a maximum induction in leaves. Moreover, the AvCAT2 expression level remains elevated after 24 h of stress application (Figure 10). The same effect was observed with AvCAT4. In contrast, AvCAT8 was down-regulated in the presence of ABA, suggesting that AvCAT8 could be a negative regulator of plant response to ABA. 

## 3. Discussion

In addition to model plants like *A. thaliana* [45], rice [11], and *N. tabacum* [20], genome-wide identification of the *CAT* gene family has also been reported in several other crops, including durum wheat [16], *G. hirsutum*, *G. max*, and *G. barbadense* [17], *H. vulgare* [46], *Z. mays* [47], pumpkin [48], bread wheat ([12], *C. sativus* [19], Rapeseed [49] and so on. Recently, sixteen *CAT* genes have been discovered and cloned from *Saccharum spontaneum* [18], five from sugarcane hybrids [28], and two *CAT* genes in *E. arundinaceus* [50]. In the present work, a total of 10 CATs encoding genes were identified by bioinformatics analysis (Table 1). According to their structure/functions, it has been shown that plant *CAT* genes are generally divided into three different groups related to photosynthetic, vascular, and reproductive functions [19,48]. The same observation was also described here; oat CAT proteins were sub-devised into three groups (Figure 1A). The same result was also described in cucumber [19]; *T. durum* [16]; and *T. aestivum* [51]. Generally, prokaryotic and eukaryotic catalases were also classified into three classes: A subgroup of bacteria and tiny plant catalase subunits were found in Clade 1. The third clade includes small subunit catalases from bacteria, fungi, protists, animals, and plants, while the second clade includes a subset of bacteria and large subunit catalases from fungi [52]. Thus, such findings strongly support the reliability of the group classifications in our study.

Catalase sequence alignment suggests a high similarity percentage (>95%) (Supplemental Figure S3) in each class. The similarity of two protein sequences in each class is very high, as previously shown for CAT identified from *T. durum* [16] and *N. plumbaginifolia* [53]. Moreover, the chromosomal localization of *AvCAT* genes was also investigated. The 10 genes were located in 7 different chromosomes. In fact, *AvCAT7* and *AvCAT10* genes were mapped in Ch6C, *AvCAT4*; *AvCAT5* and *AvCAT6* were located in Ch7D, whereas the other genes were located in the other chromosomes (Ch1D, Ch4A, Ch4C, and Chr2D) (Table 1, Figure 3). In *T. durum*, six genes were located on three different chromosomes: chromosome 6B (*TdCAT3*, *TdCAT4,* and *TdCAT6*); chromosome 4B (*TdCAT1* and *TdCAT5*) and on chromosome 6A (*TdCAT2B*) [16]. In bread wheat, 9 out of the 10 *TaCAT* genes were mapped onto the distal regions of the arms of eight different wheat chromosomes [12].

Plant CATs have two well-conserved domains. A typical plant CAT enzyme is tetrameric, and each subunit contains different conserved sequences at the catalytic site. Moreover, those proteins present a catalase activity motif (CAM) with a conserved histidine at position 65 (FARERIPERVVHARGAS), a conserved peptide sequence (PTS1) (S/E/C-K/R/H-L) at the carboxyl terminus as well as heme binding sites (HBS) containing a conserved tyrosine at position 350 (RVFAYGDTQ). The PTS1 is nine amino acids in length from the carboxy terminus and may be able to recognize the peroxisome [54]. In addition, Kamigaki et al. [55] reported the discovery of another conserved PTS1-like motif (QKL/I/V). Cucumber has four CsCAT proteins, which also present three conserved amino acids—His, Asn, and Tyr—a catalytic site—FDRERIPERVVHAKGAGA—and a conserved heme-ligand signature sequence—RLFSYNDTH. The same characteristics were also found in CAT1 isolated from durum wheat [56]. 

Protein Subcellular location is a crucial biological characteristic of proteins [57], which allows scientists to understand the mechanisms controlling protein activities. Subcellular localization of different catalase proteins was investigated in different species such as in *Arabidopsis* (the peroxisomes), in rice (peroxisomes and cytoplasm) [11], in *T. turgidum* and *T. monococcum* (TdCAT1 and TmCAT1, respectively were located in the peroxisome) [56], in bread wheat TaCAT2A/B was localized in the cytoplasm and the nucleus [12]. In this study, a PTS1-like motif (QKL/I/V) was mapped in 6 proteins (AvCAT1/2/3/5/6/7) but absent in the other proteins (Supplemental Figure S2). Interestingly, in silico analyses of those proteins, it was revealed that AvCAT1 and AvCAT2 are peroxisomal proteins. Despite the absence of a PTS1-like motif in its structure, AvCAT4 (CSS motif instead of QKL/I/V motif) is predicted to be also peroxisomal. The mechanism of peroxisomal localization of AvCAT4 remains unclear. AvCAT8 and AvCAT9 could be located in the cytoplasm, mitochondria, and peroxisome, whereas AvCAT10 is predicted to be a cytoplasmic, peroxisomal, and mitochondrial protein with a small probability of a nucleic localization. Those proteins have an MKV motif, not a PTS1-like motif (Supplemental Figure S2). Despite the presence of the PTS1-like motif, AvCAT3 is located in the nucleus, mitochondria, and cytoplasm but not in the peroxisome (Figure 5). The same finding was for AvCAT6, which has a small probability of being located in the peroxisome (Figure 5). Such findings suggest the importance of different localizations of CAT proteins in plants to eliminate the toxic H_2_O_2_ compounds. 

Moreover, the result of the *AvCAT* gene structure showed that the numbers of exons varied between two and 9, with the lowest number of exons in *AvCAT10* and the highest number in *AvCAT10*. In *T. durum*, the number of exons varies from 3 to 7 [16], whereas in *G. hirsutum*, the structure of the seven identified genes varies between seven and nine. Identified AvCAT proteins are hydrophobic (negative GRAVY index), and five proteins (AvCAT1/2/3/6/7) are thermostable (aliphatic index > 70%) (Table 2). Interestingly, the majority of identified CAT proteins in all investigated species have a negative GRAVY index, suggesting that those proteins are thermostable. Furthermore, AvCAT proteins share small, disordered regions in their sequences, and no signal peptide was mapped except for AvCAT5 (Table 2). The 2D and 3D protein structures were also studied using SOPMA and Alphafold servers, respectively. Interestingly, the structures of identified AvCAT proteins were predominantly formed by random coils (approximately half of the protein structures). Such results were also shown for durum wheat [16] and tobacco catalase proteins [20]. The AvCAT 3D models presented a variation in their structural conformation. The binding pockets play a crucial role in the protein interaction and binding sites. According to the CASTp 3.0 analysis, molecular pockets were identified on all candidates. The top three predicted pockets with the largest volume are indicated as pink, purple, and green, respectively (Figure 4). These giant pockets exhibited in the AvCAT protein structure may be related to the highest number of candidates who could bind to their atoms. Therefore, different molecular functions may be associated with these catalases in *A. sativa*.

For the diversity of protein functions in plant cell signaling, posttranslational modifications (PTMs) are significant regulators. Protein phosphorylation is a significant and well-studied PTM that affects the functionality of numerous receptors and essential elements in cellular signaling. Protein kinases and protein phosphatases, respectively, catalyze the dynamic and reversible protein phosphorylation that mostly takes place on serine (Ser) and threonine (Thr) residues in plants. In fact, many physiological processes are controlled by protein phosphorylation, such as iron uptake as Fe homeostasis is controlled by protein phosphorylation [58], nitrogen, phosphorus, and potassium uptake in plants [59], plant immunity [60], anthocyanin accumulation in apple fruits [61] and so on. As shown in Table 3, the number of phosphorylated sites in AvCAT proteins varies from 29 in AvCAT6 to 50 in AvCAT5, suggesting the importance of phosphorylation in AvCAT protein activities. Recently, it has been shown that TdCAT proteins from durum wheat presented different phosphorylation sites [16]. Moreover, TdCAT1 activity depends on the phosphorylation status of the protein. In fact, treatment of TdCAT1 by phosphatase inhibited the catalytic activity of the protein [14]. Interestingly, it has been recently shown that TdCAT1 proteins could be phosphorylated in vitro in the presence of wheat Mitogen-Activated Protein Kinase protein (TMPK3) and that the presence of TMPK3 enhanced the catalytic activity of the catalase [62]. In the current work, in silico analyses showed that AvCAT proteins could be phosphorylated by MAPKs. The importance of such results should be investigated in vivo to study the role of each phosphorylation residue in plant growth/development as well as plant response to different stresses. 

One of eukaryotic proteins’ most prevalent posttranslational modifications is glycosylation [63]. All identified CAT proteins harbor putative N-glycosylation site in their structures except for AvCAT6, which do not contain any putative glycosylation site, and AvCAT5, which harbors two putative glycosylation sites as revealed by the Protter database (Table 3). In eukaryotes, this modification regulates different signaling pathways implicated in the modulation of plant response to different stresses. In plants, the procedure of N-glycosylation keeps the chloroplast-located protein known as CAH1 stable, which plays an important function in controlling photosynthetic efficiency. The folding and transportation of proteins both benefit from N-glycosylation. Glycosylation is also crucial for stomata development. In fact, a mutation in the STT gene enhances transpiration in plants, leading to an important water loss in plants and an abnormal stomatal distribution. Thus, plants are more sensitive to drought stress [64]. Moreover, it has been shown that mutations in the N-glycosylation pathway genes *alg3-3* and *cgl1-1* in *Arabidopsis* result in a clear reduction in photosynthesis [65]. In addition, stt3a mutant plants are characterized by cell death inhibition in the presence of bir1 and bak1 serk4 mutations [66]. Finally, under-glycosylation of a β-glucosidase protein (AtBG1) inhibits ABA and Auxin biosynthesis. In fact, AtBG1 controls the transformation of conjugated IAA/ABA to active hormones, suggesting that N-glycosylation is important for stomatal development and controlling the endogen level of active hormones in response to abiotic stresses [66].

Calmodulins (CaMs), the most relevant calcium sensors, are small acidic proteins highly conserved by eukaryotes [67]. Those sensors perceive small changes in intracellular Ca^2+^ levels [68] to ensure plants’ response to different plant growth cascades and response to biotic and abiotic stresses [69,70] by fixing a large number of ligands such as transcription actors [70], MAP Kinase Phosphatase [71], pathogen-related protein (PR-1) [72]. It has been shown that durum wheat harbors 6 CATs encoding genes. All identified durum wheat CAT proteins harbor at least three conserved CaMBDs located at different portions of the proteins [16]. Moreover, we have recently characterized a conserved CaM binding domain (CaMBD) located at the C-terminal portion of the TdCAT1 protein [17] and in many other proteins such as *Arabidopsis* [73], potato [74], and sweet potato [75] TdCAT1/CaM interaction (in presence of Ca^2+^ ions) enhances the catalytic activity of the protein in Calcium dependent manner. In the present work, all CAT proteins harbor at least three calmodulin-binding domains. Those domains are located at different portions of the proteins, as previously shown for durum wheat [17] (Table 4). The results suggest that the *AvCAT* gene family has conserved catalase structural domains, presents catalytic functions, and can share the same functions as other identified plant CATs.

Gene ontology analysis was also performed. The results confirmed that all identified proteins presented catalase activity and heme/metal binding motifs, suggesting the importance of Heme and other cations in protein catalase activity. In fact, it has been recently demonstrated that durum wheat catalase activity (TdCAT1) was stimulated in the presence of Fe^2+^ and other cations (Mn^2+^; Mg^2+^; Ca^2+^; Zn^2+^; Cu^2+^). Interestingly, the catalase activity of TdCAT1 increased gradually with increasing concentrations of cations in the medium, with the most important effect being enregistered in the presence of Fe^2+^ and Mn^2+^ [13]. In addition, those proteins can interact with other proteins, as previously described, such as the CaM/Ca^2+^ complex [13] and protein kinases [62]. In addition, AvCAT1/2 and 3 are structural constituents of ribosomes, whereas AvCAT1/2 /3 and 5 have 5S rRNA binding functions (Figure 6).

Interestingly, oat CATs have different functions. In fact, all identified AvCAT control the cellular oxidant detoxification and hydrogen peroxide catabolic process. The same result was observed in durum wheat [16]. Moreover, those genes are implicated in response to hormones and different abiotic stresses. AvCAT4/5/6/7/8/9 and 10 are involved in cell response to ROSs, whereas AvCAT1/2 and three are involved in response to oxygen-containing substances, oxidative stress, intracellular nitric oxide homeostasis, Hydrogen peroxide biosynthesis processes and implicated in protein nitrosylation. In addition, AvCAT8/9 and 10 control plant response to inorganic substances and salt stress. AvCAT4/5/6 and 7 control plant response to heat, and together with AvCAT4/5 and 6, they modulate plant response to cadmium—avCAT4/5/6/7/8/9 and 10 control the circadian rhythm of the plants and their response to alcohol stress. Finally, AvCAT4/5/6/7/9 and 10 ensure plant response to acid chemicals (Figure 6).

Depending on recent bibliography research, no research has been carried out to investigate the cis-acting elements of *AvCAT* gene promoters. To further understand cis-elements of *AvCAT* genes, the 2 kb 5′ upstream region of the 10 *AvCAT* genes was analyzed using the PlantCARE database. Our results showed that all *AvCAT* genes have some basic core components. Moreover, two elements (G-box (Sp1) and ABA-response element (ABRE), which are crucial for plant response to abiotic stress, were common to all identified *AvCAT* genes (Figure 6). Such results were also observed in bread and durum wheat [12,16]. *AvCAT* genes are responsive to different abiotic stress and developmental events. For example, all *AvCAT* genes are implicated in plant response to light, whereas *AvCAT3* contains cis-elements associated with SA responses. The promoters of *AvCAT1/2/4/8* and 9 specifically contained an element related to meristem expression, suggesting that these genes may be related to meristem development. *AvCAT1/3/5/6* and 10 are responsive to Gibberellic acid, whereas *AvCAT4/5/6/7/8/9* and 10 are responsive to auxin. Moreover, *AvCAT4* and *AvCAT7* are implicated in plant response to low temperatures, and *AvCAT4/5/6/7/8/9* and 10 are responsive to drought stress. Moreover, an MYB-binding site (MBS) was found in the promoter region of oat CAT genes, which belong to classes II and III, suggesting that those *AvCATs* could be regulated by the MYB transcription factor. All those findings suggest that *AvCAT* genes can be involved in plant maturation/growth and cell differentiation by acting as ROS regulators. Moreover, our findings suggest that *AvCAT* genes of the same class may have different modes of action and that genes of different classes may work together, as previously shown for *TaCAT* and *TdCAT* genes [12,16].

Previous research demonstrated that the *CAT* gene expression patterns differed in many tissues and during various growth and development phases. In banana peel, MaCAT2 was down-regulated during fruit maturation [29]. Moreover, most of the 14 BnCATs were strongly expressed in the leaf, stem, and silique of *B. napus*. While BnCAT1, BnCAT3, and BnCAT9 did not express or were expressed at low levels in the majority of tissues, BnCAT4 and BnCAT10 were expressed at a high level [21]. Su et al. [28] and Sun et al. [76] observed that in sugarcane, ScCAT1 and ScCAT2 expressed constitutively in leaf, stem epidermal, root, stem pith, and bud, with the highest levels of ScCAT1 and ScCAT2 expression in stem epidermal and bud, respectively. CAT is crucial for growth and development, oxidative senescence, and a protective response to environmental stress in plants. Light, temperature, salt, drought, heavy metals, plant hormones, and pathogens all have an impact on CAT activity [77]. Alam and Ghosh [11] demonstrated that in *A. thaliana*, AtCAT1 expression increased in response to oxidative, drought, cold, and heat stresses, AtCAT2 expression increased in response to osmotic, drought, genotoxic, oxidative, and UV-B stresses, and AtCAT3 expression decreased in response to all stresses except cold, osmotic, and UV-B. In bananas, a strong induction of the *MaCat2* gene was detected in leaves after plant exposure to low temperatures (10 °C). This induction was low in roots [29]. A lower signal was detected in leaves when fruits were treated at 25 °C. However, in the banana fruit pulp, the *MaCat2* transcript accumulation was drastically lower at 25 °C and almost undetectable at 10 °C [29]. We observed that the *MaCat2* transcript increased in response to mechanical damage but not too high-temperature exposure (45 °C) or during fruit maturation [29]. In *Zea mays*, *ZaCat2* presented a unique function in eliminating the toxic H_2_O_2_ during senescence and regreening [78].

In this study, we investigated plant responses to different abiotic stresses. Our analyses were performed in the presence of two internal controls: ADPR and GAPDH. The *ADPR* gene has recently been demonstrated to be the best suited for all test-evaluated stresses (cold, drought, salt stress, heat stress), although the tissue determines how it expresses. This gene provided the proper regulation in roots under salt stress and in leaves under drought stress. Additionally, ADPR served as the best internal control in samples subjected to cold and heat shocks. Interestingly, authors advised caution while using actins due to their general instability [79]. Our results showed that 3 *AvCATs* were expressed constitutively in the Saudi variety’s roots, stems, and leaves. All *AvCAT* genes presented a constitutive expression under normal development conditions, with a higher expression level detected for *AvCAT1* in all tissues. *AvCAT3* gene, a member of group I, displayed a lower level of expression than other group I genes, particularly in roots and leaves, but AvCAT1 displayed a higher level of expression (Figure 8). Moreover, when the *AvCAT* gene expression patterns were analyzed, it became clear that the *AvCAT* genes belonging to the same subgroups were similarly expressed as previously expressed in many other species, such as durum wheat [16] and cotton [17]. 

Furthermore, we studied the transcript levels of the 3 *AvCATs* (*AvCAT2*, *AvCAT4,* and *AvCAT8*) genes under extreme temperatures (heat and cold) and ABA treatments and in three different tissues (Roots, Stems, and Leaves) (Figure 9 and Figure 10). Interestingly, the *AvCAT2* gene was upregulated in response to ABA but not to heat and cold stress, whereas the *AvCAT4* gene was upregulated under heat, cold, and ABA treatments, and *AvCAT8* was upregulated under heat stress conditions but downregulated in response to ABA. In bread wheat, it has been shown that *TaCAT3-A1/B/U* genes were down-regulated under cold and PEG treatments and induced under heat stress, whereas the *TaCAT2* gene was induced under heat treatment. In addition, *TaCAT3-A1/B/U* was suppressed under cold and Mannitol treatments. In durum wheat, it has been recently demonstrated that *TdCAT2* and *TdCAT3* were constrictively expressed in all tissues (Roots, Stems, and Leaves) at 10 days old stage, suggesting that these genes could play important roles in controlling durum wheat growth processes [16]. The same result was also observed in bread wheat [12]. Actually, *TaCAT2B* and *TaCAT3-A1/B/U* genes displayed constitutive patterns of expression since they were expressed in a variety of tissues at all developmental stages. Furthermore, in response to heat stress, the latter genes were activated [12]. The *CaCat2* gene was expressed throughout all of the tissues in hot peppers, whereas the *CaCat1* gene was significantly expressed in vascular tissues, and the *CaCat3* gene was constitutively expressed in young seedlings and vegetative organs although at a low level [80].

Our findings have opened up new avenues for further research and shed light on the *CAT* family genes in oats. More research is needed to fully understand the functions of the oat *AvCATs* genes. 

## 4. Materials and Methods

### 4.1. Plant Material

*A. sativa* L. seeds from the Saudi Arabian variety (cv. AlShinen) were gathered from private fields in Al-Shinen, which is east of Hai’l. Before incubation, 30 mL of a 0.5% sodium hypochlorite solution was applied to nearly 60 seeds and left on for 15 to 20 min. After that, 50 mL of sterile water was used to wash the seeds five times to eliminate the remaining sodium hypochlorite. Under 280 mol m^−2^ s^−1^ of photosynthetically active radiation and 16/8 h of light/dark conditions, incubation was carried out at 25 °C. Petri dishes (11 cm wide by 11 cm long by 2.5 cm high) with a sheet of Whatman filter paper and a piece of sponge (to conserve moisture) were used to germinate seeds. Seeds were then placed in a greenhouse.

Ten days after incubation, several stress treatments were applied to the seedlings. The stress treatments employed in this study included distilled water as a control, heat stresses (37 ° C), cold stress (4 °C), and ABA treatment (5 mM). Each treatment was carried out three times. Instantly after being harvested, the roots, stems, and leaves were frozen in liquid nitrogen and preserved at −80 °C.

### 4.2. Identification of AvCAT Gene Family

The specific conserved domains of catalase PF00199 and pfam06628 were used as query to run Blast in the genome of *A. sativa* L in Ensembl Plants release 56 (https://plants.ensembl.org/info/index.html/; accessed on 11 May 2023) [81]. The selected CAT proteins were scanned by Interpro (https://www.ebi.ac.uk/interpro/; accessed on 12 May 2023) [82], CD-Search (https://www.ncbi.nlm.nih.gov/Structure/cdd/wrpsb.cgi/; accessed on 12 May 2023) [83]; and HMMER (https://www.ebi.ac.uk/Tools/hmmer/; accessed on 12 May 2023) [84]. Thus, ten members were identified in the Avena CAT proteins family. 

### 4.3. Characterization of AvCAT Proteins and Genes

The ProtParam program [85] on the ExPASy website (https://web.expasy.org/protparam/; accessed on 13 May 2023) was used to determine the AvCAT protein’s physical and chemical characteristics, including their amino acid count, molecular weight (MW), isoelectric point (pI), hydrophobicity, and instability index. The web program SignalP-6.0 (https://services.healthtech.dtu.dk/services/SignalP-6.0/; accessed on 13 May 2023) was used to scan the existence of signal peptide in the AvCAT proteins [86]. Exon and intron distribution were performed by TbTools v1.123) [87]. MEME software (https://meme-suite.org/meme/; accessed on 16 May 2023) [88] was used to examine conserved motifs.

### 4.4. Chromosome Location and Phylogenetic Analysis of the AvCAT Gene Family

The MG2C server was used to create the chromosomal location map (http://mg2c.iask.in/mg2c_v2.1/; accessed on 25 May 2023) [89]. Using MEGA-11 software (https://www.megasoftware.net/; accessed on 17 May 2023), [90] multiple amino acid sequence alignment (MSA) was performed by MUSCLE algorithm with default settings [91] and the phylogenetic tree was inferred with the use of the maximum likelihood method with 1000 bootstrap and visualized by iTOL v 6.8 online tool (https://itol.embl.de/upload.cgi; accessed on 17 May 2023) [92].

### 4.5. The 2D and 3D Structures of Oat Catalase

Secondary structures of AvCAT proteins were predicted using SOPMA [93] (https://npsa-prabi.ibcp.fr/cgibin/npsa_automat.pl?page=npsa_sopma.html; accessed on 18 May 2023), whereas 3D structures were predicted using Alphafold online server (https://alphafold.ebi.ac.uk/; accessed on 21 May 2023) [94].

### 4.6. Gene Structure and Conserved Motifs of AvCAT Genes 

Tbtools was used to visually show the intron/exon gene organization of *AvCAT* genes [87]. The Protter database (https://wlab.ethz.ch/protter/start/; accessed on 16 May 2023) [95] was used to study the presence of signal peptides and transmembrane domains in *AvCAT* gene structures. Finally, the presence of conserved CaMBDs was identified using the Calmodulin target database (http://calcium.uhnres.utoronto.ca/ctdb/no_flash.htm; accessed on 22 May 2023) [96]. 

### 4.7. Promoter Cis-Regulatory Element Analysis of the AvCAT Gene Family

A 2 kb sequence upstream of the translation start site of *AvCAT* genes was retrieved from the *A. sativa* L. genome as the promoter sequence obtained from the Ensembl Plants database (https://plants.ensembl.org/info/index.html/; accessed on 11 May 2023) [81] and its cis-regulatory elements were predicted using PlantCARE (http://bioinformatics.psb.ugent.be/webtools/plantcare/html/; accessed on 24 May 2023) [97]. 

### 4.8. Subcellular and GO Ontology of AvCAT Proteins

Subcellular localization of AvCAT proteins was realized using Wolf PSORTserver (https://wolfpsort.hgc.jp/; accessed on 24 May 2023) [98] and visualization via Tbtools software v1.123 [81]. By PANNZER2 webtool (http://ekhidna2.biocenter.helsinki.fi/sanspanz/; accessed on 26 May 2023), Go ontology (GO) of AvCAT proteins was predicted [99].

### 4.9. RNA Extraction and Quantitative Real-Time Reverse Transcription PCR (QRT-PCR)

The RNeasy Plant Mini Kit (QIAGEN, Hilden, Germany) was used to independently extract total RNA from the roots, stems, and leaves of A. sativa L. plants (0.5 g of each tissue). After being extracted, RNA was purified from genomic DNA using an RNase-free DNase set from QIAGEN, validated on an agarose gel, quantified, and utilized for first-strand cDNA synthesis using an oligo-dT primer from the GoScript Reverse Transcription System from Promega (Madison, WI, USA). The following ingredients were used in the PCR reactions performed in 10 µL of final volume: 2 µL of cDNA (obtained from 40 ng of RNA that had undergone DNase treatment), 0.5 µL of each primer for the *AvCAT* genes at a concentration of 10 µM, 5 µL of 2 × SYBR Green I master mix, and 1 µL of RNase-free water. The reactions were constituted of a denaturation step at 95 °C for 5 min, 40 cycles of 10 s at 95 °C, 20 s at 60 °C, and 30 s at 72 °C, and a melting curve composed of 5 s at 95 °C, 1 min at 65 °C, and 5 min with an increase in temperature from 65 °C to 97 °C. Each stress condition had three biological repetitions, and each sample underwent three technological repetitions. At the conclusion of cycling, melting curve analysis was utilized to confirm whether there had been a single amplification. The triplicate PCRs’ threshold cycle (CT) values were averaged at the experiment’s conclusion and utilized for transcript quantification. Here, two internal genes were used as internal expression standards: GAPDH, and ADP-ribosyl cyclase (ADPR), the relative expression ratio of the *AvCAT* genes was computed [100] in the presence of the following primers: qADPR-F: 5′-CTCATGGTTGGTCTCGATGC-3′ and qADPR-R: 5′-ACATCCCAAACAGTGAAGCT-3′ for *ADPR* gene, and qGAPDH-F: 5′-GTTTGGCATCGTTGAGGGTT-3′ and qGAPDH-R: 5′-TGCTGCTGGGAATGATGTTG-3′ for *GAPDH* gene. Based on triplicate data, the relative expression level was determined using the 2^−∆∆CT^ formula, where ∆∆CT = (CT, Target gene CT, Actin) stressed (CT, Target gene CT, Actin). Three separate experiments (three biological replicates) with varying relative expression ratios are given.

### 4.10. Statistical Analysis

Data are reported as mean ± S.E. The results were compared statistically using the Student’s *t*-test, and differences were considered significant at *p* < 0.05.

## 5. Conclusions

In order to prevent cell death, CAT proteins act as crucial barriers by converting the harmful H_2_O_2_ into harmless components. The identification and functional characterization of the oat *AvCAT* genes is yet unknown despite the fact that *CAT* genes are essential for plant defense against various abiotic stress environments. Here, various in silico analysis approaches were looked at to improve our understanding of the CAT family in *A. sativa* plants as a whole. Based on the genome of *A. sativa* L., the ten genes that make up the *AvCAT* gene family, which has three subfamilies, were discovered in the current study. These genes were spread over seven distinct chromosomes. Other bioinformatic studies demonstrated that AvCAT proteins include highly conserved structural elements like heme-binding domains, peroxisomal targeting signal 1 (PTS1-like domains), Catalase Activity Motifs, Calmodulin binding domains, as well as Catalase-like and Catalase-related motifs. Other bioinformatic analyses demonstrated that the architectures of AvCAT proteins are substantially conserved. Additionally, examination of the *AvCAT* gene promoters revealed several cis-elements in the region upstream of the *AvCAT* genes. These components were discovered in durum wheat and may influence how the genes for growth/development, hormones, and stress responses are expressed.

## Figures and Tables

**Table 1:** Comparison features of *CAT* genes identified in *A. sativa* L.

Gene	Transcript ID	Chr	Chr Position Start…End	Strand	Gene Length (bp)	Number of Exons
*AvCAT.1*	AVESA.00001b.r3.1Dg0003456.1	1D	467,285,149… 467,289,077	forward	2357	6
*AvCAT.2*	AVESA.00001b.r3.4Ag0002488.4	4A	349,469,107… 349,472,414	forward	1881	6
*AvCAT.3*	AVESA.00001b.r3.4Cg0001036.2	4C	76,344,390… 76,347,638	reverse	1929	5
*AvCAT.4*	AVESA.00001b.r3.7Dg0000025.2	7D	1,293,105… 1,296,851	forward	1959	7
*AvCAT.5*	AVESA.00001b.r3.7Dg0002783.2	7D	468,933,423… 468,937,400	reverse	1873	8
*AvCAT.6*	AVESA.00001b.r3.7Dg0002783.1	7D	468,933,423… 468,937,398	reverse	1958	9
*AvCAT.7*	AVESA.00001b.r3.6Cg0000037.1	6C	3,581,105… 3,584,769	forward	1948	8
*AvCAT.8*	AVESA.00001b.r3.2Dg0000518.1	2D	52,851,614… 52,853,761	reverse	1807	3
*AvCAT.9*	AVESA.00001b.r3.1Ag0002627.3	1A	419,211,637… 419,213,810	forward	1823	3
*AvCAT.10*	AVESA.00001b.r3.6Cg0001322.3	6C	125,235,002… 125,237,126	reverse	2016	2

**Table 2:** Physicochemical characteristics and secondary structure prediction of AvCAT proteins were revealed by the Protparam online software (https://web.expasy.org/protparam/; accessed on 13 May 2023 and the Sopma online tool (https://npsa-prabi.ibcp.fr/cgi-bin/npsa_automat.pl?page=/NPSA/npsa_sopma.html; accessed on 18 May 2023), respectively. Alpha helix (Hh)/Beta bridge (Bb)/Extended strand (Ee)/Beta turn (Tt)/Random coil (Cc).

Protein	Protein Length (aa)	Molecular Weight (Da)	Theoretical pI:	The Instability Index (II)	Aliphatic Index	GRAVY	Hh (%)	Bb (%)	Ee (%)	Tt (%)	Cc (%)
AvCAT.1	492	56,645.85	6.54	35.40 Stable	70.35	−0.567	27.03	0	17.28	6.10	49.59
AvCAT.2	501	57,723.10	6.66	35.86 Stable	71.42	−0.555	28.34	0	16.37	5.59	49.70
AvCAT.3	546	62,231.45	7.79	42.16 Un stable	74.45	−0.481	24.54	0	15.38	4.95	55.13
AvCAT.4	496	57,157.51	6.54	31.14 Stable	68.19	−0.493	28.02	0	16.94	6.25	48.79
AvCAT.5	539	61,527.65	7.68	42.18 Un stable	69.09	−0.497	24.68	0	15.40	5.19	54.73
AvCAT.6	460	53,278.63	6.54	33.68 stable	72.04	−0.471	31.74	0	14.57	6.52	47.17
AvCAT.7	533	61,126.44	6.88	34.90 stable	71.33	−0.430	25.70	0	15.57	5.07	53.66
AvCAT.8	494	57,009.54	6.53	31.62 stable	66.46	−0.554	27.73	0	13.65	5.87	52.83
AvCAT.9	494	57,039.57	6.53	32.34 Stable	66.46	−0.555	26.11	0	14.78	6.07	53.04
AvCAT.10	494	57,034.55	6.42	33.14 Stable	66.86	−0.537	27.13	0	15.18	5.67	50.02

**Table 3:** General characteristics of AvCAT proteins using Protparam and Protter online software 1.0. (https://wlab.ethz.ch/protter/start/; accessed on 16 May 2023).

Gene Name	Length (aa)	N-Glycosylation Site	Number of Phosphorylated Sites	Disordered Regions	Signal Peptide
*AvCAT1*	492	N-247	38	420–440	No
*AvCAT2*	501	N-256	39	415–435	No
*AvCAT3*	546	N-299	41	460–500	No
*AvCAT4*	496	N-28	38	1–20	No
*AvCAT5*	539	N-35N-75	50	20–70	Yes 24 aa (N-terminal region)
*AvCAT6*	460	--	29	--	No
*AvCAT7*	533	N-69	39	25–40	No
*AvCAT8*	494	N-247	34	5–27 404–423	No
*AvCAT9*	494	N-247	36	5–27 404–423	No
*AvCAT10*	494	N-247	37	5–27 404–423	No

**Table 4:** Number of identified CaMBDs in AvCAT proteins.

Gene Name	Number of Putative CaMBDs	Typical CaMBD	Position	IQ Motif	Position
*AvCAT-1*	4	3	58–79; 207–229; 462–486	1	296–315
*AvCAT-2*	4	3	67–88; 214–238; 471–499	1	305–324
*AvCAT-3*	4	3	112–133; 259–283; 516–544	1	350–369
*AvCAT-4*	3	2	58–79; 204–224	1	296–315
*AvCAT-5*	3	2	106–125; 251–271	1	343–362
*AvCAT-6*	3	2	26–47; 172–192	1	264–283
*AvCAT-7*	3	2	99–120; 235–265	1	337–356
*AvCAT-8*	4	3	48–78; 204–227; 341–361	1	296–315
*AvCAT-9*	4	3	48–78; 204–227; 341–361	1	296–315
*AvCAT10*	4	3	48–78; 204–227; 341–361	1	296–315

## Data Availability

The data generated and analyzed during this study are included in this article.

## Supplementary Materials

The following supporting information can be downloaded at https://www.mdpi.com/article/10.3390/plants12213694/s1. Figure S1: Phylogenetic tree of the catalase proteins in *A. sativa* generated by MEGA 11 with maximum-likelihood; Figure S2: Alignment of AvCAT proteins by MUSCLE algorithm using MEGA 11 program. The conserved CAM motif is marked with a red rectangle, and the Histidine residue is marked with a “*”; the PTS1 motif is marked with a blue rectangle, and the heme-binding domain is marked by a black rectangle with the conserved Y350 residue; Figure S3: 2D structures of the ten identified AvCATs in oat (*A. sativa* L.). Different structures are represented, such as the Alpha helix (blue), Extended strand (red), Beta turn (green), and Random coil (purple).

## References

[1] Tuteja N. (2007). Mechanisms of High Salinity Tolerance in Plants. Methods Enzymol..

[2] Hirayama T., Shinozaki K. (2010). Research on Plant Abiotic Stress Responses in the Post-genome Era: Past, Present and Future. Plant J..

[3] Vellosillo T., Vicente J., Kulasekaran S., Hamberg M., Castresana C. (2010). Emerging Complexity in Reactive Oxygen Species Production and Signaling during the Response of Plants to Pathogens. Plant Physiol..

[4] Sharma P., Jha A.B., Dubey R.S., Pessarakli M. (2012). Reactive Oxygen Species, Oxidative Damage, and Antioxidative Defense Mechanism in Plants under Stressful Conditions. J. Bot..

[5] Bose J., Rodrigo-Moreno A., Shabala S. (2014). ROS Homeostasis in Halophytes in the Context of Salinity Stress Tolerance. J. Exp. Bot..

[6] Bhattacharjee S. (2012). The Language of Reactive Oxygen Species Signaling in Plants. J. Bot..

[7] Karuppanapandian T., Moon J.-C., Kim C., Manoharan K., Kim W. (2011). Reactive Oxygen Species in Plants: Their Generation, Signal Transduction, and Scavenging Mechanisms. Aust. J. Crop Sci..

[8] Noctor G., Mhamdi A., Chaouch S., Han Y.I., Neukermans J., Marquez-Garcia B., Queval G., Foyer C.H. (2012). Glutathione in Plants: An Integrated Overview. Plant. Cell Environ..

[9] Shangari N., O’Brien P.J. (2006). Catalase Activity Assays. Curr. Protoc. Toxicol..

[10] Borges P.T., Frazao C., Miranda C.S., Carrondo M.A., Romão C. (2014). V Structure of the Monofunctional Heme Catalase DR 1998 from D Einococcus Radiodurans. FEBS J..

[11] Alam N.B., Ghosh A. (2018). Comprehensive Analysis and Transcript Profiling of Arabidopsis Thaliana and Oryza Sativa Catalase Gene Family Suggests Their Specific Roles in Development and Stress Responses. Plant Physiol. Biochem..

[12] Zhang Y., Zheng L., Yun L., Ji L., Li G., Ji M., Shi Y., Zheng X. (2022). Catalase (CAT) Gene Family in Wheat (*Triticum aestivum* L.): Evolution, Expression Pattern and Function Analysis. Int. J. Mol. Sci..

[13] Ghorbel M., Feki K., Tounsi S., Haddaji N., Hanin M., Brini F. (2022). The Activity of the Durum Wheat (*Triticum durum* L.) Catalase 1 (TdCAT1) Is Modulated by Calmodulin. Antioxidants.

[14] Ghorbel M., Feki K., Tounsi S., Bouali N., Besbes M., Brini F. (2022). The Putative Auto-Inhibitory Domain of Durum Wheat Catalase (TdCAT1) Positively Regulates Bacteria Cells in Response to Different Stress Conditions. Antioxidants.

[15] Ghorbel M., Besbes M., Haddaji N., Bouali N., Brini F. (2022). Identification and Expression Profiling of Two Saudi Arabia Catalase Genes from Wheat and Barley in Response to Abiotic and Hormonal Stresses. Antioxidants.

[16] Ghorbel M., Zribi I., Besbes M., Bouali N., Brini F. (2023). Catalase Gene Family in Durum Wheat: Genome-Wide Analysis and Expression Profiling in Response to Multiple Abiotic Stress Conditions. Plants.

[17] Wang W., Cheng Y., Chen D., Liu D., Hu M., Dong J., Zhang X., Song L., Shen F. (2019). The Catalase Gene Family in Cotton: Genome-Wide Characterization and Bioinformatics Analysis. Cells.

[18] Wu Q., Chen Y., Zou W., Pan Y.-B., Lin P., Xu L., Grisham M.P., Ding Q., Su Y., Que Y. (2023). Genome-Wide Characterization of Sugarcane Catalase Gene Family Identifies a ScCAT1 Gene Associated Disease Resistance. Int. J. Biol. Macromol..

[19] Hu L., Yang Y., Jiang L., Liu S. (2016). The Catalase Gene Family in Cucumber: Genome-Wide Identification and Organization. Genet. Mol. Biol..

[20] Liu Z., Wang D., Tang H., Li H., Zhang X., Dong S., Zhang L., Yang L. (2023). Identification and Analysis of the Catalase Gene Family Response to Abiotic Stress in *Nicotiana tabacum* L.. Agronomy.

[21] Raza A., Su W., Gao A., Mehmood S.S., Hussain M.A., Nie W., Lv Y., Zou X., Zhang X. (2021). Catalase (CAT) Gene Family in Rapeseed (*Brassica napus* L.): Genome-Wide Analysis, Identification, and Expression Pattern in Response to Multiple Hormones and Abiotic Stress Conditions. Int. J. Mol. Sci..

[22] Sharma S., Hooda V. (2018). Identification of Coding Sequence and Its Use for Functional and Structural Characterization of Catalase from *Phyllanthus emblica*. Bioinformation.

[23] Scandalios J.G., Acevedo A., Ruzsa S. (2000). Catalase Gene Expression in Response to Chronic High Temperature Stress in Maize. Plant Sci..

[24] Zang Y., Liu J., Tang X.X., Zhou B. (2018). Description of a Zostera Marina Catalase Gene Involved in Responses to Temperature Stress. PeerJ.

[25] Lin K.-H., Huang H.-C., Lin C.-Y. (2010). Cloning, Expression and Physiological Analysis of Broccoli Catalase Gene and Chinese Cabbage Ascorbate Peroxidase Gene under Heat Stress. Plant Cell Rep..

[26] Cansev A., Gulen H., Eris A. (2011). The Activities of Catalase and Ascorbate Peroxidase in Olive (*Olea europaea* L. Cv. Gemlik) under Low Temperature Stress. Hortic. Environ. Biotechnol..

[27] Gao J.-J., Tao L.I., YU X. (2009). Gene Expression and Activities of SOD in Cucumber Seedlings Were Related with Concentrations of Mn^2+^, Cu^2+^, or Zn^2+^ under Low Temperature Stress. Agric. Sci. China.

[28] Su Y., Guo J., Ling H., Chen S., Wang S., Xu L., Allan A.C., Que Y. (2014). Isolation of a Novel Peroxisomal Catalase Gene from Sugarcane, Which Is Responsive to Biotic and Abiotic Stresses. PLoS ONE.

[29] Figueroa-Yáñez L., Cano-Sosa J., Castaño E., Arroyo-Herrera A.-L., Caamal-Velazquez J.H., Sanchez-Teyer F., López-Gómez R., De Los Santos-Briones C., Rodríguez-Zapata L. (2012). Phylogenetic Relationships and Expression in Response to Low Temperature of a Catalase Gene in Banana (*Musa acuminata* Cv.‘“Grand Nain”’) Fruit. Plant Cell Tissue Organ Cult. (PCTOC).

[30] Harker K.N., O’donovan J.T. (2013). Editorial Recent Weed Control, Weed Management, and Integrated Weed Management. Weed Technol..

[31] Prates L.L., Yu P. (2017). Recent research on inherent molecular structure, physiochemical properties, and bio-functions of food and feed-type Avena sativa oats and processing-induced changes revealed with molecular microspectroscopic techniques. Appl. Spectrosc. Rev..

[32] Butt M.S., Tahir-Nadeem M., Khan M.K.I., Shabir R., Butt M.S. (2008). Oat: Unique among the cereals. Eur. J. Nutr..

[33] Bai J., Yan W., Wang Y., Yin Q., Liu J., Wight C., Ma B. (2018). Screening Oat Genotypes for Tolerance to Salinity and Alkalinity. Front. Plant Sci..

[34] Heuschele D.J., Case A., Smith K.P. (2019). Evaluation of Fast Generation Cycling in Oat (*Avena sativa*). Cereal Res. Commun..

[35] Goff S.A., Ricke D., Lan T.H., Presting G., Wang R., Dunn M., Glazebrook J., Sessions A., Oeller P., Varma H. (2002). A draft sequence of the rice genome (*Oryza sativa* L. ssp. japonica). Science.

[36] Schnable P.S., Ware D., Fulton R.S., Stein J.C., Wei F., Pasternak S., Liang C., Zhang J., Fulton L., Graves T.A. (2009). The B73 maize genome: Complexity, diversity, and dynamics. Science.

[37] Appels R., Eversole K., Feuillet C., Keller B., Rogers J., Stein N., Pozniak C.J., Choulet F., Distelfeld A., Poland J. (2018). Shifting the limits in wheat research and breeding using a fully annotated reference genome. Science.

[38] Forsberg R.A., Reeves D.L. (1992). Breeding Oat Cultivars for Improved Grain Quality. Oat Sci. Technol..

[39] Wang B., Song F.B. (2006). Physiological Responses and Adaptive Capacity of Oats to Saline-Alkali Stress. Ecol. Environ. Ecol. Env..

[40] Storsley J., Jew S., Ames N. (2013). Health Claims for Oat Products: A Global Perspective. Oats Nutrition and Technology.

[41] Kamal N., Tsardakas Renhuldt N., Bentzer J., Gundlach H., Haberer G., Juhász A., Lux T., Bose U., Tye-Din J.A., Lang D. (2022). The Mosaic Oat Genome Gives Insights into a Uniquely Healthy Cereal Crop. Nature.

[42] Islam M.R., Xue X., Mao S., Ren C., Eneji A.E., Hu Y. (2011). Effects of Water-saving Superabsorbent Polymer on Antioxidant Enzyme Activities and Lipid Peroxidation in Oat (*Avena sativa* L.) under Drought Stress. J. Sci. Food Agric..

[43] Willenborg C.J., Wildeman J.C., Miller A.K., Rossnagel B.G., Shirtliffe S.J. (2005). Oat Germination Characteristics Differ among Genotypes, Seed Sizes, and Osmotic Potentials. Crop Sci..

[44] Singh R., De S., Belkheir A. (2013). Avena Sativa (Oat), a Potential Neutraceutical and Therapeutic Agent: An Overview. Crit. Rev. Food Sci. Nutr..

[45] Du Y., Wang P., Chen J., Song C. (2008). Comprehensive Functional Analysis of the Catalase Gene Family in Arabidopsis Thaliana. J. Integr. Plant Biol..

[46] Skadsen R.W., Schulze-Lefert P., Herbst J.M. (1995). Molecular Cloning, Characterization and Expression Analysis of Two Catalase Isozyme Genes in Barley. Plant Mol. Biol..

[47] Wu Z.-X., Xu N.-W., Yang M., Li X.-L., Han J.-L., Lin X.-H., Yang Q., Lv G.-H., Wang J. (2022). Responses of Photosynthesis, Antioxidant Enzymes, and Related Gene Expression to Nicosulfuron Stress in Sweet Maize (*Zea mays* L.). Environ. Sci. Pollut. Res..

[48] Esaka M., Yamada N., Kitabayashi M., Setoguchi Y., Tsugeki R., Kondo M., Nishimura M. (1997). CDNA Cloning and Differential Gene Expression of Three Catalases in Pumpkin. Plant Mol. Biol..

[49] Raza A. (2021). Eco-Physiological and Biochemical Responses of Rapeseed (*Brassica napus* L.) to Abiotic Stresses: Consequences and Mitigation Strategies. J. Plant Growth Regul..

[50] Liu Y., Hu X., Yao Y., Xu L., Xing S. (2016). Isolation and Expression Analysis of Catalase Genes in Erianthus Arundinaceus and Sugarcane. Sugar Tech.

[51] Tyagi S., Singh K., Upadhyay S.K. (2021). Molecular Characterization Revealed the Role of Catalases under Abiotic and Arsenic Stress in Bread Wheat (*Triticum aestivum* L.). J. Hazard. Mater..

[52] Klotz M.G., Klassen G.R., Loewen P.C. (1997). Phylogenetic Relationships among Prokaryotic and Eukaryotic Catalases. Mol. Biol. Evol..

[53] Willekens H., Langebartels C., Tire C., Van Montagu M., Inze D., Van Camp W. (1994). Differential Expression of Catalase Genes in *Nicotiana plumbaginifolia* (L.). Proc. Natl. Acad. Sci. USA.

[54] Chen J., Wang G., Cheng S. (2008). Progress about Catalase Function in Plant Stress Reactions. Acta Bot. Boreali-Occident. Sin..

[55] Kamigaki A., Mano S., Terauchi K., Nishi Y., Tachibe-Kinoshita Y., Nito K., Kondo M., Hayashi M., Nishimura M., Esaka M. (2003). Identification of Peroxisomal Targeting Signal of Pumpkin Catalase and the Binding Analysis with PTS1 Receptor. Plant J..

[56] Tounsi S., Kamoun Y., Feki K., Jemli S., Saïdi M.N., Ziadi H., Alcon C., Brini F. (2019). Localization and Expression Analysis of a Novel Catalase from Triticum Monococcum TmCAT1 Involved in Response to Different Environmental Stresses. Plant Physiol. Biochem..

[57] Ang L.-H., Chattopadhyay S., Wei N., Oyama T., Okada K., Batschauer A., Deng X.-W. (1998). Molecular Interaction between COP1 and HY5 Defines a Regulatory Switch for Light Control of Arabidopsis Development. Mol. Cell.

[58] Li W., Han X., Lan P. (2022). Emerging Roles of Protein Phosphorylation in Plant Iron Homeostasis. Trends Plant Sci..

[59] Hao D., Li X., Kong W., Chen R., Liu J., Guo H., Zhou J. (2023). Phosphorylation Regulation of Nitrogen, Phosphorus, and Potassium Uptake Systems in Plants. Crop J..

[60] Lin H., Wang M., Chen Y., Nomura K., Hui S., Gui J., Zhang X., Wu Y., Liu J., Li Q. (2022). An MKP-MAPK Protein Phosphorylation Cascade Controls Vascular Immunity in Plants. Sci. Adv..

[61] Xing Y., Sun W., Sun Y., Li J., Zhang J., Wu T., Song T., Yao Y., Tian J. (2023). MPK6-mediated HY5 Phosphorylation Regulates Light-induced Anthocyanin Accumulation in Apple Fruit. Plant Biotechnol. J..

[62] Ghorbel M., Haddaji N., Feki K., Tounsi S., Chihaoui M., Alghamdi A., Mseddi K., Brini F. (2023). Identification of a Putative Kinase Interacting Domain in the Durum Wheat Catalase 1 (TdCAT1) Protein. Heliyon.

[63] Wang Y., Tan J., Sutton-Smith M., Ditto D., Panico M., Campbell R.M., Varki N.M., Long J.M., Jaeken J., Levinson S.R. (2001). Modeling a Human Genetic Disease Involving a Glycosylation Defect in the Mouse: Conservation of N-Glycan Function and Insights into CDG-IIa Pathogenesis. Glycobiology.

[64] Jiao Q., Chen T., Niu G., Zhang H., Zhou C., Hong Z. (2020). N-Glycosylation Is Involved in Stomatal Development by Modulating the Release of Active Abscisic Acid and Auxin in Arabidopsis. J. Exp. Bot..

[65] Jiao Q.-S., Niu G.-T., Wang F.-F., Dong J.-Y., Chen T.-S., Zhou C.-F., Hong Z. (2020). N-Glycosylation Regulates Photosynthetic Efficiency of Arabidopsis Thaliana. Photosynthetica.

[66] de Oliveira M.V.V., Xu G., Li B., de Souza Vespoli L., Meng X., Chen X., Yu X., de Souza S.A., Intorne A.C., de A. (2016). Manhães, A.M.E. Specific Control of Arabidopsis BAK1/SERK4-Regulated Cell Death by Protein Glycosylation. Nat. Plants.

[67] Snedden W.A., Fromm H. (2001). Calmodulin as a Versatile Calcium Signal Transducer in Plants. New Phytol..

[68] Kudla J., Batistič O., Hashimoto K. (2010). Calcium Signals: The Lead Currency of Plant Information Processing. Plant Cell.

[69] Dubrovina A.S., Aleynova O.A., Ogneva Z.V., Suprun A.R., Ananev A.A., Kiselev K. (2019). V The Effect of Abiotic Stress Conditions on Expression of Calmodulin (CaM) and Calmodulin-like (CML) Genes in Wild-Growing Grapevine Vitis Amurensis. Plants.

[70] Iqbal Z., Shariq Iqbal M., Singh S.P., Buaboocha T. (2020). Ca2+/Calmodulin Complex Triggers CAMTA Transcriptional Machinery under Stress in Plants: Signaling Cascade and Molecular Regulation. Front. Plant Sci..

[71] Ghorbel M., Zaidi I., Robe E., Ranty B., Mazars C., Galaud J.-P., Hanin M. (2015). The Activity of the Wheat MAP Kinase Phosphatase 1 Is Regulated by Manganese and by Calmodulin. Biochimie.

[72] Ghorbel M., Zribi I., Missaoui K., Drira-Fakhfekh M., Azzouzi B., Brini F. (2021). Differential Regulation of the Durum Wheat Pathogenesis-Related Protein (PR1) by Calmodulin TdCaM1. 3 Protein. Mol. Biol. Rep..

[73] Yang T., Poovaiah B.W. (2002). Hydrogen Peroxide Homeostasis: Activation of Plant Catalase by Calcium/Calmodulin. Proc. Natl. Acad. Sci. USA.

[74] Afiyanti M., Chen H.-J. (2014). Catalase Activity Is Modulated by Calcium and Calmodulin in Detached Mature Leaves of Sweet Potato. J. Plant Physiol..

[75] Chen H.-J., Wu S.-D., Huang G.-J., Shen C.-Y., Afiyanti M., Li W.-J., Lin Y.-H. (2012). Expression of a Cloned Sweet Potato Catalase SPCAT1 Alleviates Ethephon-Mediated Leaf Senescence and H_2_O_2_ Elevation. J. Plant Physiol..

[76] Sun T., Liu F., Wang W., Wang L., Wang Z., Li J., Que Y., Xu L., Su Y. (2018). The Role of Sugarcane Catalase Gene ScCAT2 in the Defense Response to Pathogen Challenge and Adversity Stress. Int. J. Mol. Sci..

[77] Song X.H., Zhao F.Y. (2007). Research Progress on Catalase in Plants. J Anhui Agric. Sci.

[78] Lino-Neto T., Piques M.C., Barbeta C., Sousa M.F., Tavares R.M., Pais M.S. (2004). Identification of Zantedeschia Aethiopica Cat1 and Cat2 Catalase Genes and Their Expression Analysis during Spathe Senescence and Regreening. Plant Sci..

[79] Tajti J., Pál M., Janda T. (2021). Validation of Reference Genes for Studying Different Abiotic Stresses in Oat (*Avena sativa* L.) by RT-qPCR. Plants.

[80] Lee S.H., An C.S. (2005). Differential Expression of Three Catalase Genes in Hot Pepper (*Capsicum annuum* L.). Mol. Cells.

[81] Yates A.D., Allen J., Amode R.M., Azov A.G., Barba M., Becerra A., Bhai J., Campbell L.I., Carbajo Martinez M., Chakiachvili M. (2022). Ensembl Genomes 2022: An Expanding Genome Resource for Non-Vertebrates. Nucleic Acids Res..

[82] Paysan-Lafosse T., Blum M., Chuguransky S., Grego T., Pinto B.L., Salazar G.A., Bileschi M.L., Bork P., Bridge A., Colwell L. (2023). InterPro in 2022. Nucleic Acids Res..

[83] Lu S., Wang J., Chitsaz F., Derbyshire M.K., Geer R.C., Gonzales N.R., Gwadz M., Hurwitz D.I., Marchler G.H., Song J.S. (2020). CDD/SPARCLE: The Conserved Domain Database in 2020. Nucleic Acids Res..

[84] Potter S.C., Luciani A., Eddy S.R., Park Y., Lopez R., Finn R.D. (2018). HMMER Web Server: 2018 Update. Nucleic Acids Res..

[85] Garg V.K., Avashthi H., Tiwari A., Jain P.A., Ramkete P.W., Kayastha A.M., Singh V.K. (2016). MFPPI–multi FASTA ProtParam interface. Bioinformation.

[86] Teufel F., Almagro Armenteros J.J., Johansen A.R., Gíslason M.H., Pihl S.I., Tsirigos K.D., Nielsen H. (2022). SignalP 6.0 predicts all five types of signal peptides using protein language models. Nat. Biotechnol..

[87] Chen C., Chen H., Zhang Y., Thomas H.R., Frank M.H., He Y., Xia R. (2020). TBtools: An integrative toolkit developed for interactive analyses of big biological data. Mol. Plant.

[88] Bailey T.L., Boden M., Buske F.A., Frith M., Grant C.E., Clementi L., Ren J., Li W.W., Noble W.S. (2009). MEME SUITE: Tools for Motif Discovery and Searching. Nucleic Acids Res..

[89] Chao J., Li Z., Sun Y., Aluko O.O., Wu X., Wang Q., Liu G. (2021). MG2C: A User-Friendly Online Tool for Drawing Genetic Maps. Mol. Hortic..

[90] Tamura K., Stecher G., Kumar S. (2021). MEGA11: Molecular evolutionary genetics analysis version 11. Mol. Biol. Evol..

[91] Edgar R.C. (2004). MUSCLE: A Multiple Sequence Alignment Method with Reduced Time and Space Complexity. BMC Bioinform..

[92] Letunic I., Bork P. (2021). Interactive Tree Of Life (ITOL) v5: An Online Tool for Phylogenetic Tree Display and Annotation. Nucleic Acids Res..

[93] Geourjon C., Deleage G. (1995). SOPMA: Significant improvements in protein secondary structure prediction by consensus prediction from multiple alignments. Bioinformatics.

[94] Jumper J., Evans R., Pritzel A., Green T., Figurnov M., Tunyasuvunakool K., Hassabis D. (2020). AlphaFold 2. Fourteenth Critical Assessment of Techniques for Protein Structure Prediction.

[95] Omasits U., Ahrens C.H., Müller S., Wollscheid B. (2014). Protter: Interactive protein feature visualization and integration with experimental proteomic data. Bioinformatics.

[96] Yap K.L., Kim J., Truong K., Sherman M., Yuan T., Ikura M. (2000). Calmodulin Target Database. J. Struct. Funct. Genom..

[97] Lescot M., Déhais P., Thijs G., Marchal K., Moreau Y., Van de Peer Y., Rouzé P., Rombauts S. (2002). PlantCARE, a Database of Plant Cis-Acting Regulatory Elements and a Portal to Tools for in Silico Analysis of Promoter Sequences. Nucleic Acids Res..

[98] Horton P., Park K.-J., Obayashi T., Fujita N., Harada H., Adams-Collier C.J., Nakai K. (2007). WoLF PSORT: Protein Localization Predictor. Nucleic Acids Res..

[99] Törönen P., Holm L. (2022). PANNZER—A Practical Tool for Protein Function Prediction. Protein Sci..

[100] Livak K.J., Schmittgen T.D. (2001). Analysis of Relative Gene Expression Data Using Real-Time Quantitative PCR and the 2^−ΔΔCT^ Method. Methods.

