# Supplementary material for: Genome-Wide Investigation and Expression Analysis of the Catalase Gene Family in Oat Plants (Avena sativa L.)
Source: Plants (Basel). 2023 Oct 26;12(21):3694. doi: 10.3390/plants12213694 (PMC10650400; doi:10.3390/plants12213694)
Supplement: Supplementary file 1 [file plants-12-03694-s001.zip › plants-2601677-supplementary.pptx]

## Slide 1
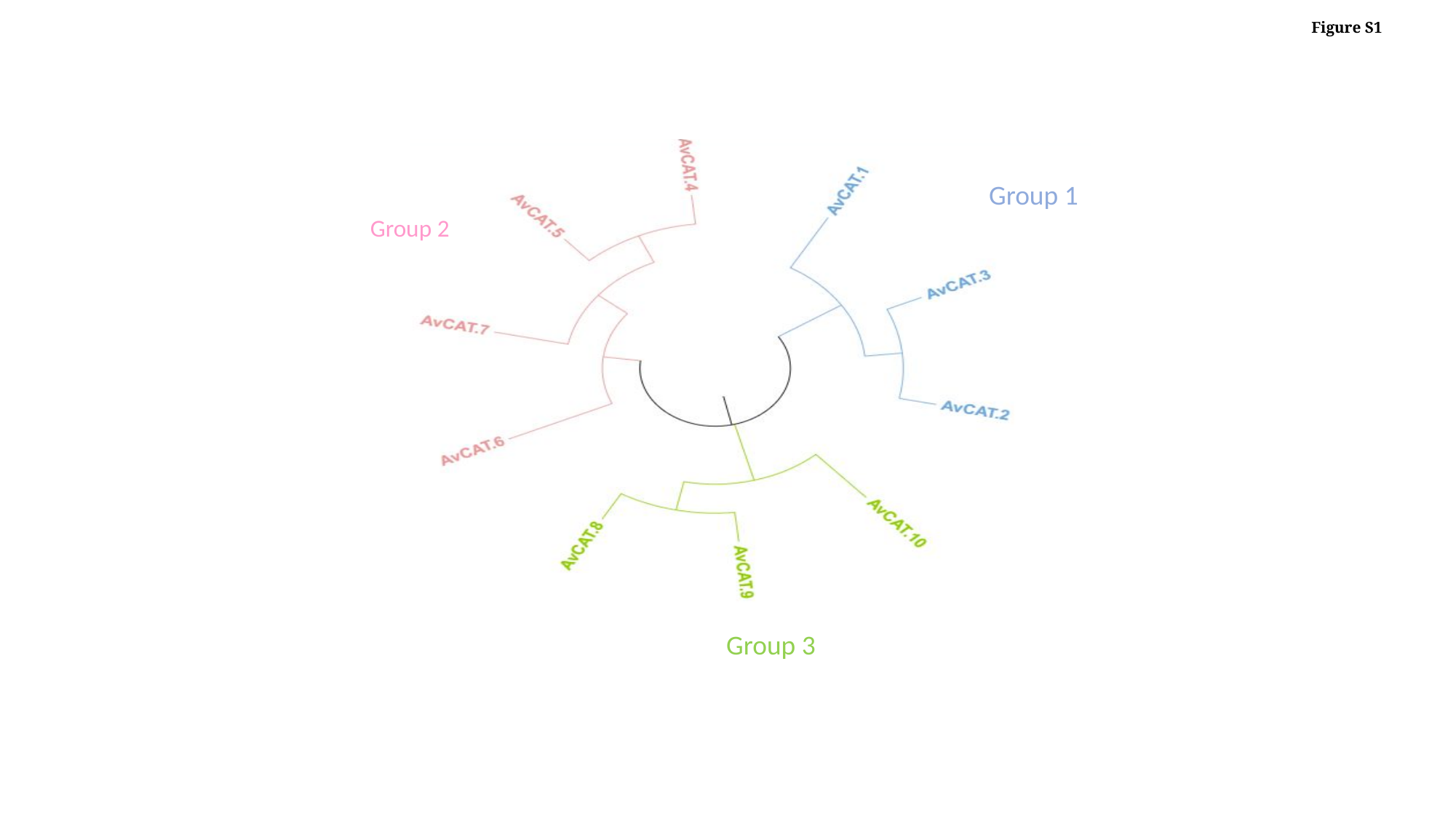

Figure S1
Group 1
Group 2
Group 3

## Slide 2
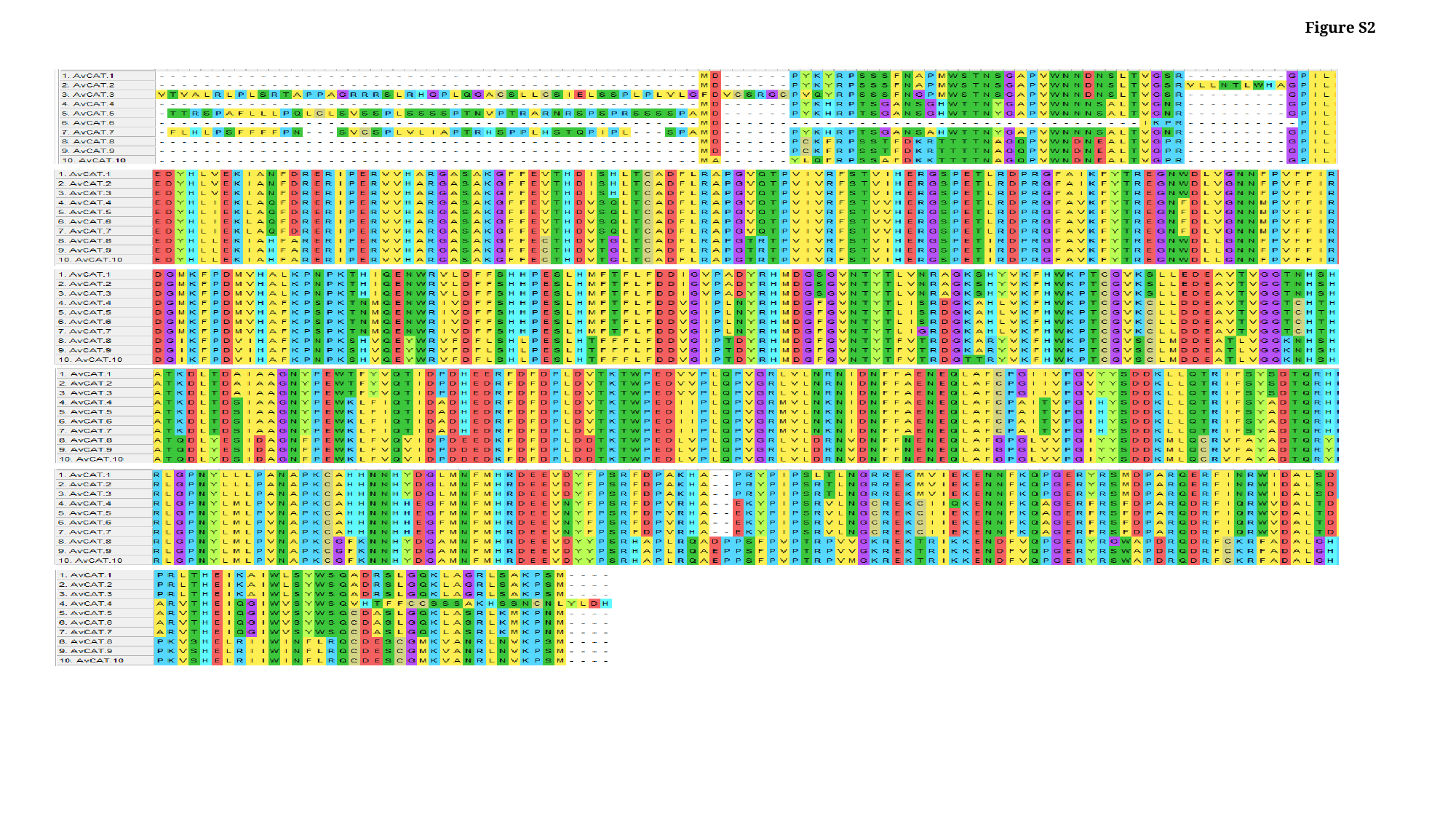

Figure S2

## Slide 3
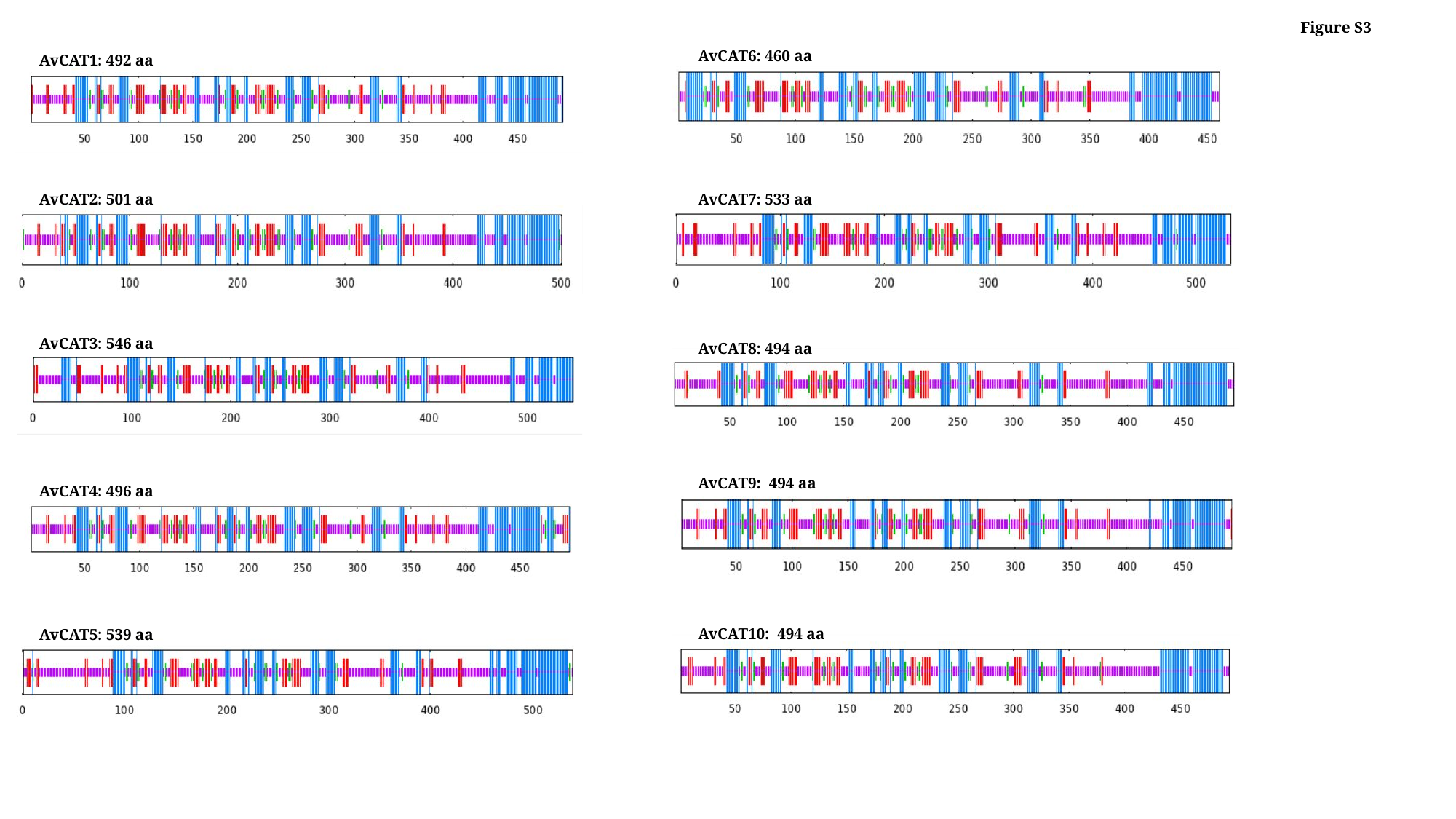

Figure S3
AvCAT6: 460 aa
AvCAT1: 492 aa
AvCAT2: 501 aa
AvCAT7: 533 aa
AvCAT3: 546 aa
AvCAT8: 494 aa
AvCAT9: 494 aa
AvCAT4: 496 aa
AvCAT10: 494 aa
AvCAT5: 539 aa
